# Xanthone-1,2,4-triazine and Acridone-1,2,4-triazine Conjugates: Synthesis and Anticancer Activity

**DOI:** 10.3390/ph16030403

**Published:** 2023-03-07

**Authors:** Sougata Santra, Ainur D. Sharapov, Ramil F. Fatykhov, Anastasya P. Potapova, Igor A. Khalymbadzha, Maria I. Valieva, Dmitry S. Kopchuk, Grigory V. Zyryanov, Alexander S. Bunev, Vsevolod V. Melekhin, Vasiliy S. Gaviko, Andrey A. Zonov

**Affiliations:** 1 Department of Organic and Biomolecular Chemistry, Ural Federal University, Mira 19, 620002 Ekaterinburg, Russia; 2 Medicinal Chemistry Center, Togliatti State University, Belorusskaya 14, 445020 Togliatti, Russia; 3 Department of Medical Biology and Genetics, Ural State Medical University, Repina 3, 620028 Ekaterinburg, Russia; 4 M.N. Mikheev Institute of Metal Physics, Ural Branch of the Russian Academy of Sciences, Kovalevskoy Street 18, 620108 Ekaterinburg, Russia

**Keywords:** xanthone, acridone, 1,2,4-triazine, colorectal cancer HCT116, glioblastoma A-172, breast ductal carcinoma Hs578T

## Abstract

A total of 21 novel xanthone and acridone derivatives were synthesized using the reactions of 1,2,4-triazine derivatives with 1-hydroxy-3-methoxy-10-methylacridone, 1,3-dimethoxy-, and 1,3-dihydroxanthone, followed by optional dihydrotiazine ring aromatization. The synthesized compounds were evaluated for their anticancer activity against colorectal cancer HCT116, glioblastoma A-172, breast cancer Hs578T, and human embryonic kidney HEK-293 tumor cell lines. Five compounds (**7a**, **7e**, **9e**, **14a**, and **14b**) displayed good in vitro antiproliferative activities against these cancer cell lines. Compounds **7a** and **7e** demonstrated low toxicity for normal human embryonic kidney (HEK-293) cells, which determines the possibility of further development of these compounds as anticancer agents. Annexin V assay demonstrated that compound **7e** activates apoptotic mechanisms and inhibits proliferation in glioblastoma cells.

## 1. Introduction

Cancer, a malignant cell growth, is the second-leading cause of death in the world [1]. Annually, about 19 million people are diagnosed with cancer worldwide, leading to approximately 10 million deaths [1]. The constantly emerging incidence of cancer worldwide makes oncological diseases one of the most serious public health problems. Therefore, exploration and development of novel anticancer drugs are crucial tasks of medicinal chemistry.

**Figure 1:**
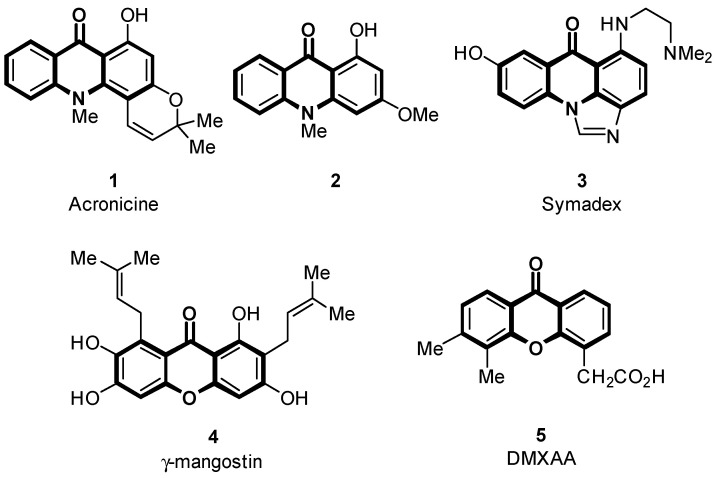
Some important acridone and xanthone derivatives.

Xanthone and acridone are privileged scaffolds for searching antitumor activity (Figure 1), and they are found in both natural and non-natural anticancer compounds. For example, Acronicine **1**, a known naturally occurring anti-tumor agent, was identified as an agent having activity against human colon carcinoma HCT116 [2], human prostate carcinoma DU145 [2], human epidermoid carcinoma KB-3-1 [3], and other cell lines [3,4,5,6,7].

A precursor in the acronicine biosynthesis is 3-Methoxy-1-hydroxy-9-methylacridone **2** [8] and it occurs in many plant species [9,10,11,12,13,14]. This compound demonstrates activity on HeLa [14] and colorectal cancer HCT116 [15] cells, human leukemic lymphoblasts CCRF-CEM, glioblastoma cell line U-87, and human alveolar basal epithelial adenocarcinoma A549 [16] cell lines. Symadex **3**, also known as C-1311, is a synthetic antitumor agent that passed Phase II clinical trials [17]. Previously, it was shown experimentally that during the initial stage of its action, C-1311 forms stable intercalation complexes with DNA duplexes [17].

Xanthone, a dibenzo-γ-pyrone, may be considered an oxa counterpart of acridone. Xanthone is another framework that is well known for its anti-cancer properties [18]. The anticancer activity of this heterocyclic system is mediated by multiple targets: caspase activation, RNA binding, DNA cross-linking, kinase, aromatase, and topoisomerase inhibition. 

There are also many compounds with anticancer activity among xanthones. For example, it was demonstrated the anticancer activity of α-, β-, and γ-mangostin, and mangiferin, a natural product isolated from the fruit and *Garcinia mangostana* (*Clusiaceae*).

DMXAA is an example of a synthetic xanthone with antitumor activity [19] mediated by vascular collapse and tumor necrosis through tumor necrosis factor-α induction. DMXAA exhibits low toxicity to normal cells with suitable pharmacokinetic parameters. It successfully passed phase II clinical trials but was withdrawn from phase III clinical trials due to insufficient effectiveness in humans. 

Thus, acridone and xanthone derivatives are privileged frameworks for the development of anticancer agents. However, anticancer agents currently under development that contain these heterocycles have some drawbacks. They are not always sufficiently active and may have poor solubility [20]. At the same time, the synthesis of modified analogues of natural acridones and xanthones is an effective method to optimize their antitumor properties [21]. Therefore, the synthesis of new acridone and xanthone derivatives is an urgent task in the search for new anticancer compounds.

## 2. Results and Discussion

In the continuation of our previous work on the synthesis of acridone analogues [22], we have obtained new derivatives of xanthone and acridone and tested them for their activity against several cancer cell lines.

### 2.1. Synthesis of Novel Acridone and Xanthone Derivatives

#### 2.1.1. Synthesis of Acridone Derivatives

We chose 1-hydroxy-3-methoxyacridone **2** as a convenient starting material that has potential for solubility due to the presence of a hydroxyl group, has a low molecular weight, and was easily available through the condensation of anthranilic acid with phloroglucinol and subsequent N,O-dimethylation [23]. Thus, this acridone can be modified to obtain substances with the expected acceptable pharmacokinetic properties. 

**Scheme 1:**
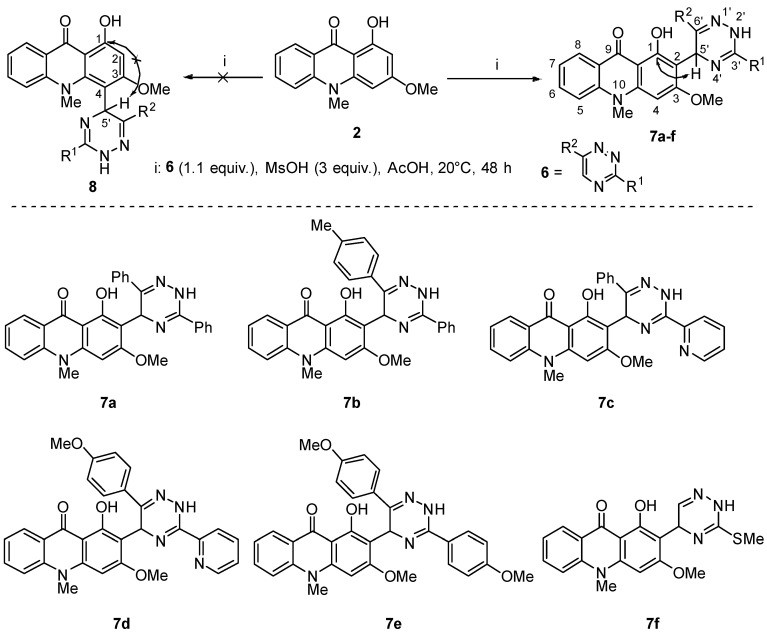
Synthesis of dihydrotriazine-substituted acridones **7a**–**f**.

The reaction of 1-hydroxy-3-methoxy-9-methylacridone **2** with the triazines **6a**–**f** in acetic acid in the presence of methanesulfonic acid provided compounds **7a**–**f** in good yields (Scheme 1). 

The structure of compounds **7a**–**f** is consistent with their NMR spectral data. The ^13^C NMR spectra of compounds **7a**–**f** comprise the characteristic resonances for the dihydrotriazine C5′ sp^3^ carbon atom at 45 ppm, while the resonance of hydrogen at this sp^3^ carbon is observed at 6.3–6.4 ppm in the ^1^H NMR spectra. In order to exclude the alternative structure of the isomeric C4 substituted product **8**, 2D ^1^H–^13^C HMBC spectra for compound **7f** were recorded. In the HMBC spectra, the signals of the C1 atom at 161.6 has cross-peak with the C5′proton resonance at 6.3–6.4 ppm (compound **7**, Scheme 1). In the case of an alternative substitution at the carbon atom in position 4 (compound **8**, Scheme 1), this cross-peak cannot be observed since the dihydrotriazine fragment is too far from the carbon atom C1.

**Scheme 2:**
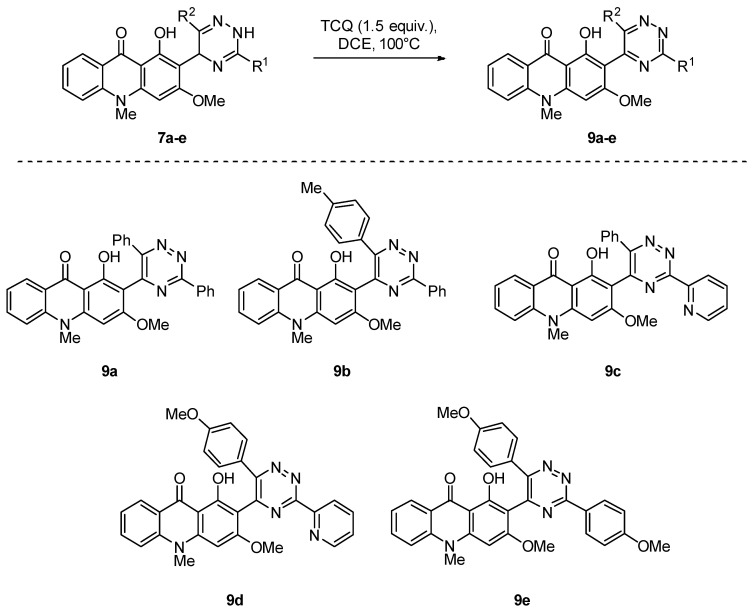
Oxidation of dihydrotriazines **7a**–**e** to triazines **9a**–**e**.

We further modified the compounds **7a**–**e** by oxidizing the dihydrotriazine ring to a fully aromatic triazine (Scheme 2). Oxidation was performed by boiling a dichloroethane solution of compound **7** with tetrachloro-*p*-benzoquinone (TCQ). During the oxidation, the sp^3^-carbon atom signal shifts downfield in the ^13^C spectrum, while the hydrogen atom signal (δ = 6.3–6.4 ppm) at this carbon disappears in the ^1^H NMR spectrum. The oxidation had no significant effect on the biological activity, as demonstrated using the MTT assay (Table 1, compound **9e**), but the solubility of the aromatized triazines **9a**–**e** in aqueous medium was significantly impaired by the oxidation.

**Scheme 3:**
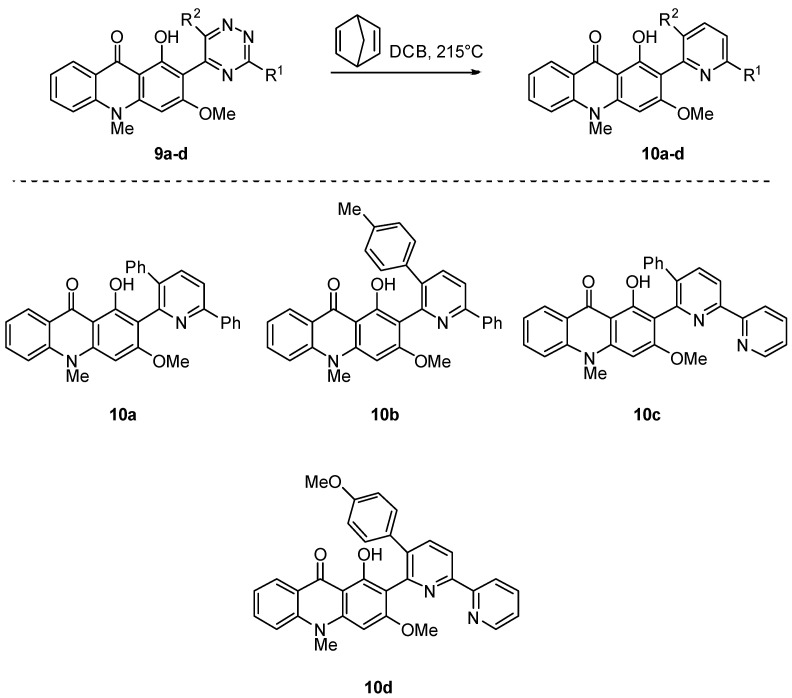
Synthesis of pyridines **10a**–**d** from triazines **9a**–**d**.

**Figure 2:**
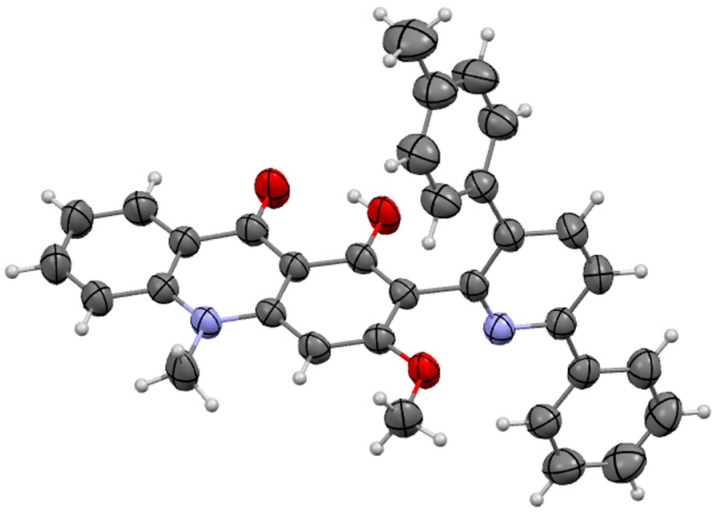
X-ray crystal structure of pyridine **10b** (CCDC 2231907).

To further evaluate the role of the 1,2,4-triazine ring for anticancer activity, we transformed the 1,2,4-triazine moiety of compounds **9a**–**d** into pyridine compounds **10a**–**d** (Scheme 3). The reaction proceeds as [2+4] cycloaddition followed by nitrogen and cyclopentadiene extrusion (Boger reaction). We used previously described conditions suitable for this transformation [24,25]. The structure of compounds **10** is consistent with their NMR spectral data. The structure of the product **10b** was proven using X-ray diffraction analysis (Figure 2). 

#### 2.1.2. Synthesis of Xanthone Derivatives

Further, we modified compound **7a** by replacing the NMe moiety with an oxygen atom in order to study the role and influence of the nitrogen atom in the tricyclic dibenzoannulated scaffold of the obtained compounds.

**Scheme 4:**
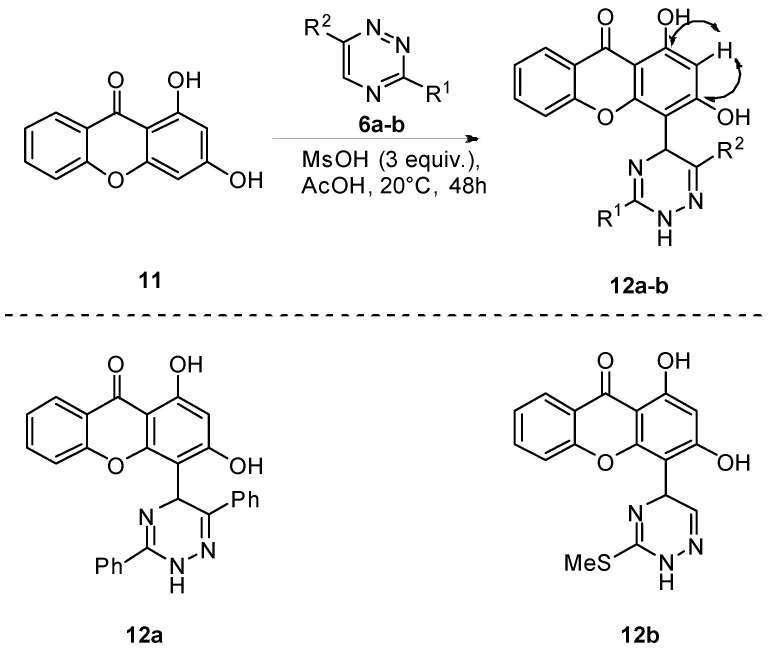
Synthesis of dihydrotriazine-substituted 1,3-dihydroxyxanthones **12a**–**b**.

The reaction of 1,3-dihydroxyxanthone **11** with 3,5-diphenyl- and 3-methylthiotriazines **6a** and **6b** provides dihydrotriazines **12a** and **12b**, respectively (Scheme 4). To our surprise, the reaction proceeded at position 4 of 1,3-dihydroxyxanthone, not at position 2 as observed for acridones (Scheme 1). The position of addition of the triazine ring was supported by the HMBC ^1^H-^13^C spectrum of compound **12b**, wherein cross-peaks between the proton at C2 (δ = 6.3 ppm) and C1 and C3 carbons are registered (Scheme 4). We can attribute the different regiochemistry of the triazine addition to the influence of the steric factor, which, in the presence of the N-methyl group, directs the reaction to the sterically less difficult position 2.

**Scheme 5:**
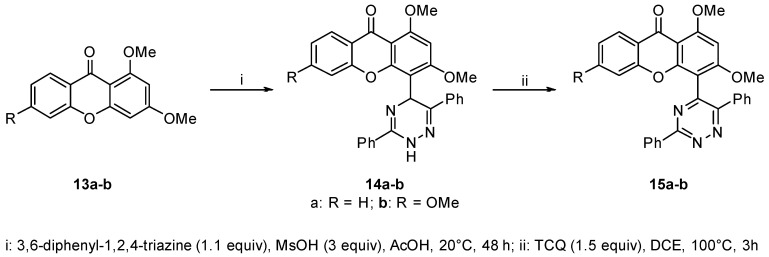
Synthesis of dihydrotriazine-substituted 1,3-dihydroxyxanthones **14a**–**b** and **15a**–**b**.

We also obtained xanthones in which both the hydroxy groups were replaced with methoxy groups (Scheme 5) via the reaction of xanthone **11** with triazine **6a**. The dihydrotriazines **14a**–**b** obtained in this scheme were oxidized to triazines **15a**–**b** in a similar manner as described earlier (Scheme 2 and Scheme 4).

### 2.2. Anticancer Activity of the Obtained Acridone and Xanthone Compounds

Acridones **7a**–**e** were tested for their antiproliferative activity by using the MTT (3-(4,5-dimethylthiazol-2-yl)-2,5-diphenyltetrazolium bromide) assay [26,27]. The results of a single-concentration screening against human colorectal cancer cells (HCT116) are presented in Table 1. As evident from the screening results, compounds **7b** and **7d** exhibited no antiproliferative activity against the HCT116 cancer cell line. On the other hand, derivatives **7a**, **7c,** and **7e** revealed profound antiproliferative action. We tested promising compounds **7a** and **7e** on other cancer lines, glioblastoma A-172 and breast ductal carcinoma Hs-578T, and found high activity of the tested compounds on these cell lines. In addition, an evaluation of the action of compounds **7a** and **7e** on normal human embryonic kidney (HEK-293) cells showed that these compounds demonstrated low toxicity and, as a result, good therapeutic indexes (IC_50_ (HEK-293)/IC_50_ (cancer) = 10–27) on the cancer cell lines studied.

It is interesting to note that the introduction of the triazine ring significantly increases the anticancer activity of the acridone pharmacophore in some cases (Table 1, cf. compound **2** and compounds **7a**, **7e**, and **9e**). However, in some cases, the activity does not change significantly (Table 1, compound **7b**).

It is important to note the critical role of the triazine cycle for antitumor activity. Thus, the transformation of the triazine ring into pyridine results in the complete disappearance of activity (compounds **7a** and **7c** vs. **10a** and **10c**, Table 1).

Xanthone derivatives **14a**–**b** showed moderate activity in the HCT116 cancer cell test, while the aromatic triazine **15b** and dihydroxy counterparts **12a** and **12b** showed no activity (Table 1).

### 2.3. Investigation of the Anticancer Mechanism of Action of Acridone Compounds

A highly specific and sensitive method for detecting apoptosis is a method based on the treatment of cells with fluorescently-tagged Annexin V (FITC) proteins. Annexin V is able to bind to phosphatidylserines that appear on the cell surface during apoptosis. Additionally, propidium iodide (PI) dye was used to stain DNA inside dead cells or those with irreversibly damaged membranes (necrotic or late apoptotic cells).

**Figure 3:**
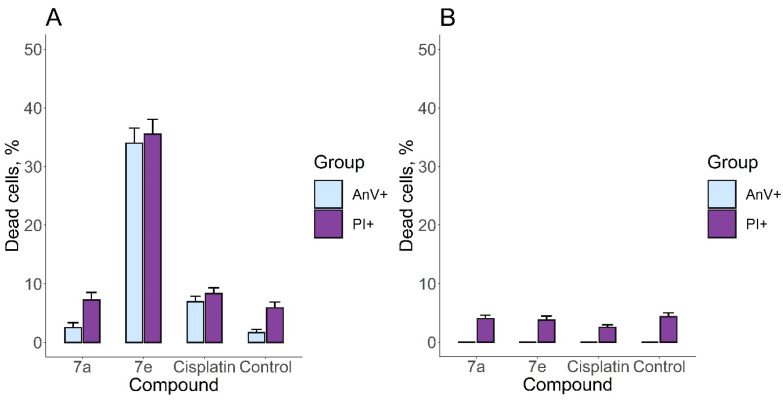
Comparison of the proportion of apoptotic (Annexin V+/PI-) and other dead cells (PI+) in glioblastoma A-172 (**A**) and human embryonic kidney HEK-293 (**B**) cultures, mean ± CI-95%.

Based on the results of cell staining with a mixture of Annexin V-FITC and PI, the number of A-172 and HEK-293 live, apoptotic, and dead cells was calculated for the compounds that showed the greatest effectiveness in suppressing the viability of malignant cells, **7a** and **7e** (Figure 3).

**Figure 4:**
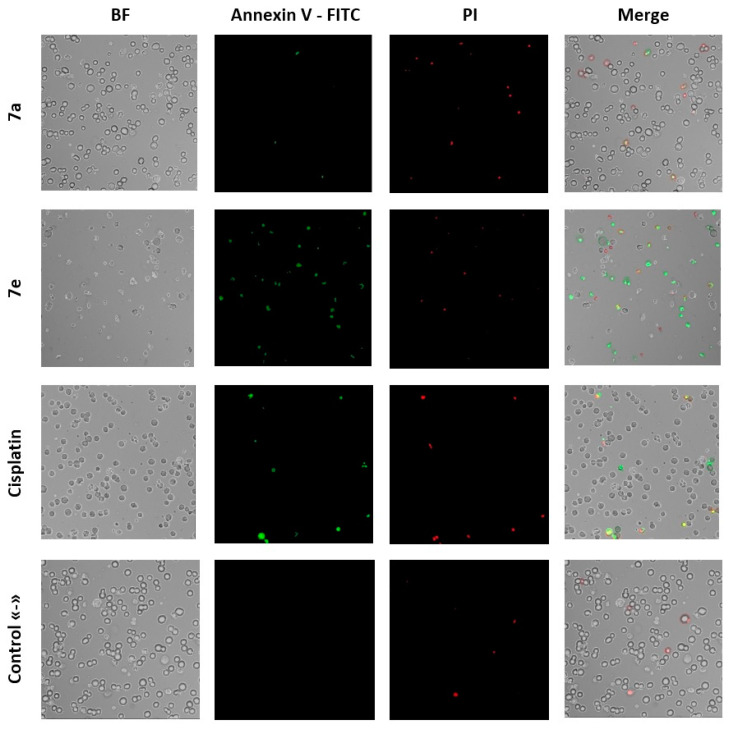
Fluorescence staining of human glioblastoma A-172 cells with Annexin V-FITC and PI dyes under the influence of the test substances **7a** and **7e** compared to cisplatin (6 µM concentration) and intact cells. Cells in early apoptosis stained green (Annexin V-FITC), cells in late apoptosis and necrosis stained red (PI), 200× magnification.

**Figure 5:**
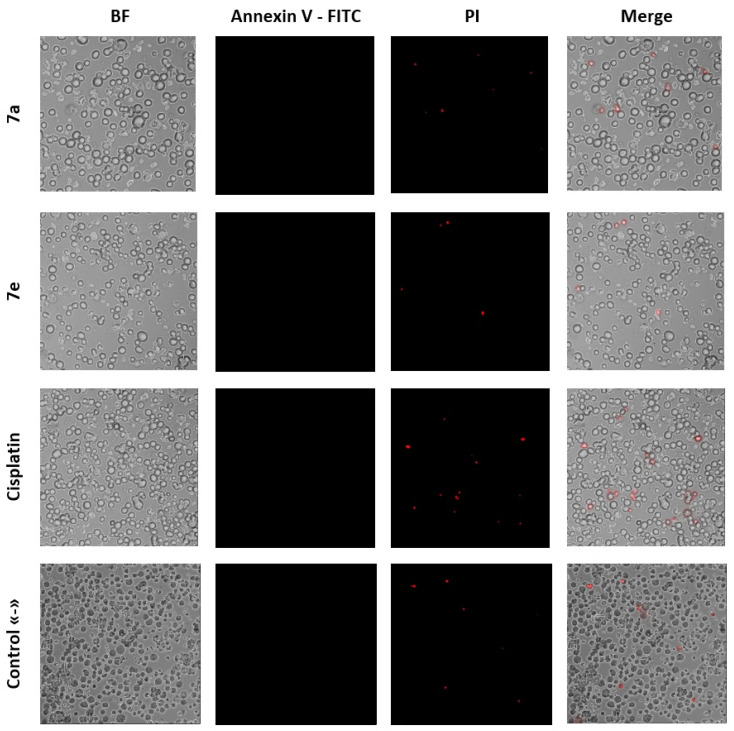
Fluorescence staining of human embryonic HEK-293 kidney cells with Annexin V-FITC and PI dyes under the action of the test compounds **7a** and **7e** in comparison with cisplatin (concentration of 6 µM) and intact cells. Cells in early apoptosis were stained green (Annexin V-FITC), cells in late apoptosis and necrosis were stained red (PI), 200× magnification.

The data obtained confirm the marked effect of **7e** against malignant cells (Figure 4), using the A-172 line as an example. In addition, the effect of the studied compounds was compared in the experiment with the widely used anticancer drug cisplatin at a similar concentration (6 µM) to compare the intensity of the cytotoxic effect. Compound **7e** showed a more intense effect against tumor cells of the A-172 line than cisplatin. At the same time, there was no significant increase in the number of dead cells in the HEK-293 line (Figure 3 and Figure 5).

Measurement of the cell’s ability to synthesize DNA is one of the most important methods for evaluating cell proliferative activity, determining toxicity, and evaluating antitumor drugs. The EdU (5-ethyl-2’-deoxyuridine) assay is a nucleoside analog of thymidine and is incorporated into DNA during active DNA synthesis. The detection is based on a copper-catalyzed covalent reaction between azide and alkyne, which are contained in the dye and EdU, respectively.

**Figure 6:**
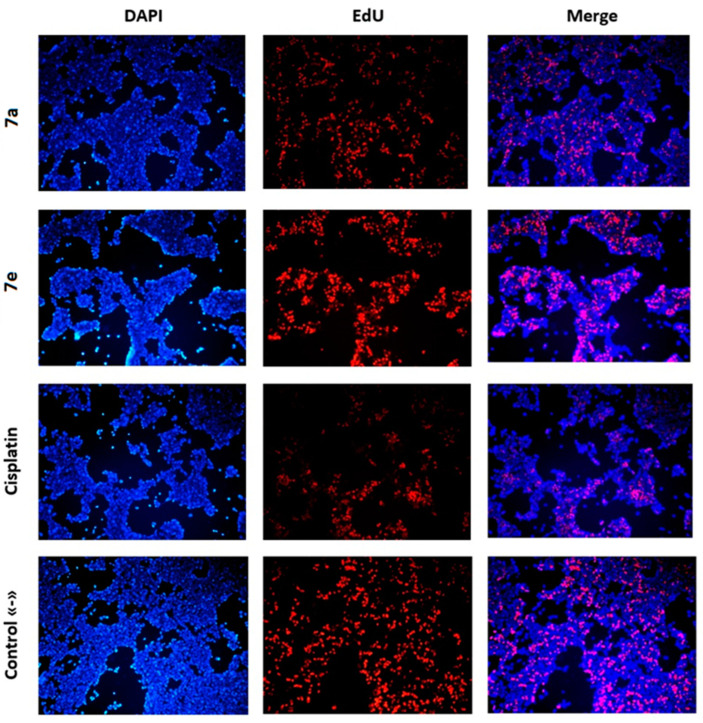
Assessment of DNA synthesis intensity in human embryonic kidney cell cultures of HEK-293 line under the action of compounds **7a** and **7e**. Blue color stains the nuclei of all cells using DAPI dye, and red nuclei of cells with active DNA synthesis (EdU)*,* at 100× magnification.

**Figure 7:**
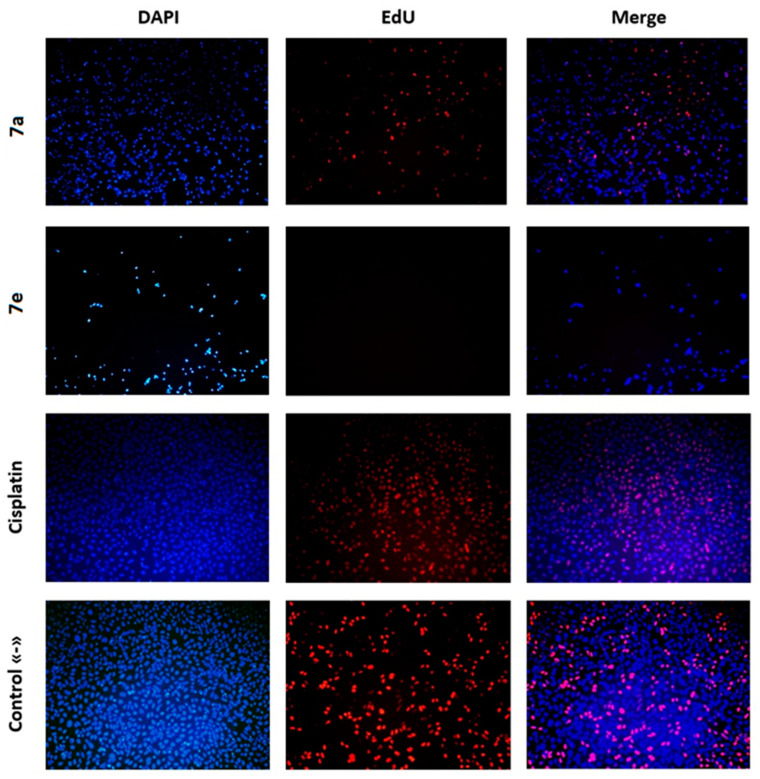
Assessment of DNA synthesis intensity in human glioblastoma cell cultures of the A-172 line under the influence of compounds **7a** and **7e**. Blue color stains the nuclei of all cells using DAPI dye, and red nuclei of cells with active DNA synthesis (EdU), at 100× magnification.

Assessing the effect of compounds **7a** and **7e** under study on the ability of cells to synthesize DNA in the HEK-293 line (Figure 6), a minimal effect comparable to intact cells, which served as a negative control, can be noted. Some decrease in proliferative activity can be noted in cisplatin-treated cells. At the same time, a more pronounced effect of compounds **7a** and **7e** on DNA synthesis in cells is observed in line A-172 (Figure 7). It should also be noted that no signs of cell proliferation were detected in the experiment with compound **7e**. In addition, Figure 4 and Figure 7 show a significant decrease in the total number of cells in the experiment with compound **7e** relative to the other groups.

Thus, the results obtained indicate the activation of apoptotic mechanisms and the inhibition of proliferation in glioblastoma cells under the influence of compound **7e**. Taken together, this may indicate a pronounced selective antitumor effect of compound **7e**.

## 3. Materials and Methods

### 3.1. Synthesis of Novel Acridone and Xanthone Compounds

All chemicals were purchased from commercial sources and used as received, unless otherwise specified. Column chromatography was performed on SiO_2_ (230–400 mesh). TLC plates coated with SiO_2_ 60F_254_ were visualized under UV light.

^1^H NMR and ^13^C{1H} NMR spectra were recorded at room temperature on a BRUKER AVANCE 400 spectrometer (^1^H, 400 MHz) or on a BRUKER NEO 600 spectrometer (^1^H, 600 MHz) in DMSO-d_6_, DMF-d_7_, or CDCl_3_ as the deuterated solvent. Chemical shifts were reported in the δ scale relative to the residual DMSO-d_6_, DMF-d_7_, or CDCl_3_ solvent peaks (2.50 ppm and 39.52 ppm for ^1^H and ^13^C, respectively for DMSO-d_6_, 8.03, and 163.15 ppm for ^1^H and ^13^C, respectively for DMF-d_7_, and 7.26 and 77.17 for ^1^H and ^13^C, respectively for CDCl_3_). NMR spectra were registered at ambient temperature.

Structural studies were performed using equipment available in the Collaborative Access Center “Testing Center of Nanotechnology and Advanced Materials” at the Mikheev Institute of Metal Physics, Ural Branch, Russian Academy of Sciences. The X-ray diffraction analysis was performed at room temperature on a Rigaku XtaLAB Synergy-S diffractometer. The calculations were performed using Olex 2 v. 171.41.120a 64-bit software.

According to the published procedures, 1-hydroxy-3-methoxy-10-methylacridin-9(10*H*)-one **2** [23], 1,2,4-triazines **6** [27], and xanthones **11** and **13** [28] were prepared.

#### 3.1.1. Synthesis of Compounds **7a**–**7f**

In acetic acid (10 mL), 1-hydroxy-3-methoxy-10-methylacridin-9(10*H*)-one **2** (257 mg, 1.0 mmol), 1,2,4-triazine **6** (1.1 mmol), and methanesulfonic acid (288 mg, 3.0 mmol) were dissolved and the mixture allowed to stand for 48 h. The mixture was poured in water and quenched with an NaHCO_3_ solution, and the precipitate was filtered and recrystallized from ethyl acetate yielding pure **7a**–**7f**.

2-(3,6-Diphenyl-2,5-dihydro-1,2,4-triazin-5-yl)-1-hydroxy-3-methoxy-10-methylacridin-9(10*H*)-one **7a**. Yellow powder, m.p. = 220–222 °C. 56% yield. ^1^H NMR (400 MHz, DMSO-d_6_) δ 15.70 (s, 1H), 10.83–11.14 (br s, 1H), 8.17–8.26 (m, 1H), 7.67–7.95 (m, 6H), 7.21–7.54 (m, 7H), 6.40–6.49 (br s, 2H), 3.90 (s, 3H), 3.77 (s, 3H). ^13^C NMR (101 MHz, DMSO-d_6_) δ 179.6, 164.3, 161.2, 149.0, 143.6, 141.6, 139.3, 136.1, 134.2, 133.5, 130.1, 128.5, 128.2 (2C), 128.1 (2C), 126.1 (2C), 125.3 (2C), 124.8, 121.6, 119.8, 115.9, 110.6, 103.9, 87.6, 63.5, 55.9, 34.0. Anal. Calcd for C_30_H_24_N_4_O_3_: C, 73.76; H, 4.95; N, 11.47%. Found: C, 73.66; H, 5.02; N, 11.30%. 

1-Hydroxy-3-methoxy-10-methyl-2-(3-phenyl-6-(*p*-tolyl)-2,5-dihydro-1,2,4-triazin-5-yl)acridin-9(10*H*)-one **7b**. Yellow powder, m.p. = 235–237 °C. 80% yield. ^1^H NMR (400 MHz, DMSO-d_6_) δ 15.63 (s, 1H), 10.89 (s, 1H), 8.21–8.30 (m, 1H), 7.73–7.90 (m, 4H), 7.65 (d, *J* = 8.0 Hz, 2H), 7.37–7.50 (m 3H), 7.27–7.37 (m, 1H), 7.08 (d, *J* = 8.0 Hz, 2H), 6.48 (s, 1H), 6.37 (s, 1H), 3.90 (s, 3H), 3.81 (s, 3*H*), 2.20 (s, 3H). ^13^C NMR (101 MHz, DMSO-d_6_) δ 179.7, 164.3, 161.2, 149.0, 143.6, 141.7, 139.2, 138.0, 134.3, 133.6, 133.3, 130.0, 128.7 (2C), 128.1 (2C), 126.1 (2C), 125.4, 124.8 (2C), 121.7, 119.9, 116.0, 110.8, 104.0, 87.6, 56.0, 45.4, 34.1, 20.7. Anal. Calcd for C_31_H_26_N_4_O_3_: C, 74.09; H, 5.21; N, 11.15%. Found: C, 74.22; H, 5.49; N, 10.93%.

1-Hydroxy-3-methoxy-10-methyl-2-(6-phenyl-3-(pyridin-2-yl)-2,5-dihydro-1,2,4-triazin-5-yl)acridin-9(10*H*)-one **7c**. Yellow powder, m.p. > 250 °C. 96% yield. ^1^H NMR (400 MHz, DMSO-d_6_) δ 15.63 (s, 1H), 10.29–10.90 (br s, 1H), 8.60–8.68 (m, 1H), 8.23–8.31 (m, 1H), 7.99–8.06 (m, 1H), 7.70–7.91 (m, 5H), 7.46–7.55 (m, 1H), 7.19–7.38 (m, 4H), 6.50 (s, 1H), 6.44 (s, 1H), 3.90 (s, 3H), 3.82 (s, 3H). ^13^C NMR (101 MHz, DMSO-d_6_) δ 179.6, 164.2, 161.3, 149.9, 148.0, 147.5, 143.7, 141.6, 140.0, 136.9, 135.8, 134.2, 128.4, 127.9 (2C), 125.3, 125.2, 124.9 (2C), 121.5, 120.6, 119.8, 115.8, 110.1, 103.9, 87.6, 55.9, 45.3, 34.0. Anal. Calcd for C_29_H_23_N_5_O_3_S: C, 71.15; H, 4.74; N, 14.31%. Found: C, 71.32; H, 4.99; N, 14.04%.

1-Hydroxy-3-methoxy-2-(6-(4-methoxyphenyl)-3-(pyridin-2-yl)-2,5-dihydro-1,2,4-triazin-5-yl)-10-methylacridin-9(10*H*)-one **7d**. Yellow powder, m.p. > 250 °C. 89% yield. ^1^H NMR (400 MHz, DMSO-d_6_) δ 15.62 (br s, 1H), 10.59 (s, 1H), 8.63–8.64 (m, 1H), 8.28–8.30 (m, 1H), 7.99–8.01 (m, 1H), 7.83–7.87 (m, 1H), 7.79–7.80 (m, 2H), 7.70 (d, *J* = 8.4 Hz, 2H), 7.48–7.51 (m, 1H), 7.32–7.36 (m, 1H), 6.84 (d, *J* = 8.4 Hz, 2H), 6.52 (s, 1H), 6.40 (s, 1H), 3.91 (s, 3H), 3.85 (s, 3H), 3.69 (s, 3H). ^13^C NMR (101 MHz, DMSO-d_6_) δ 179.6, 164.2, 161.2, 159.5, 150.1, 148.0, 147.4, 143.6, 141.7, 139.4, 136.8, 134.2, 128.3, 126.3 (2C), 125.3, 125.0, 121.5, 120.4, 119.8, 115.8, 113.4 (2C), 110.5, 104.0, 87.7, 55.9, 54.9, 45.7, 34.0. Anal. Calcd for C_30_H_25_N_5_O_4_: C, 69.35; H, 4.85; N, 13.48%. Found: C, 69.36; H, 4.97; N. 13.23%.

2-(3,6-bis(4-Methoxyphenyl)-2,5-dihydro-1,2,4-triazin-5-yl)-1-hydroxy-3-methoxy-10-methylacridin-9(10*H*)-one **7e**. Yellow powder, m.p. = 252–253 °C. 78% yield. ^1^H NMR (400 MHz, DMSO-d_6_) δ 15.63 (br s, 1H), 10.75 (s, 1H), 8.30–8.32 (m, 1H), 7.81–7.86 (m, 2H), 7.78 (d, *J* = 8.6 Hz, 2H), 7.70 (d, *J* = 8.5 Hz, 2H), 7.35–7.39 (m, 1H), 6.94 (d, *J* = 8.5 Hz, 2H), 6.84 (d, *J* = 8.6 Hz, 2H), 6.55 (s, 1H), 6.31 (s, 1H), 3.92 (s, 3H), 3.87 (s, 3H), 3.78 (s, 3H), 3.68 (s, 3H). ^13^C NMR (101 MHz, DMSO-d_6_) δ 179.8, 164.4, 161.0, 160.6, 159.5, 148.5, 143.7, 141.8, 138.9, 134.4, 128.6, 127.5 (2C), 126.2 (2C), 125.9, 125.5, 121.7, 119.9, 116.1, 113.6 (2C), 113.4 (2C), 111.0, 104.1, 87.8, 56.1, 55.2, 55.0, 45.3, 34.3. Anal. Calcd for C_32_H_28_N_4_O_5_: C, 70.06; H, 5.14; N, 10.21%. Found: C, 69.91; H, 5.03; N, 10.35%.

1-Hydroxy-3-methoxy-10-methyl-2-(3-(methylthio)-2,5-dihydro-1,2,4-triazin-5-yl)acridin-9(10*H*)-one **7f**. Yellow powder, m.p. = 215–220 °C dec. 85% yield. ^1^H NMR (400 MHz, DMSO-d_6_) δ 15.32 (s, 1H), 10.48 (s, 1H), 8.21–8.23 (m, 1H), 7.82 (br s, 2H), 7.31–7.35 (m, 1H), 6.75 (s, 1H), 6.52 (s, 1H), 4.82 (s, 1H), 3.93 (s, 3H), 3.86 (s, 3H), 2.26 (s, 3H). ^13^C NMR (101 MHz, DMSO-d_6_) δ 179.6, 164.1, 161.9, 153.0, 143.9, 141.7, 140.6, 134.3, 125.5, 121.7, 119.9, 116.0, 107.3, 104.1, 87.6, 56.1, 48.9, 34.2, 12.7. Anal. Calcd for C_19_H_18_N_4_O_3_S: C, 59.67; H, 4.74; N, 14.65%. Found: C, 59.58; H, 4.90; N, 14.47%.

#### 3.1.2. Synthesis of Compounds **9a**–**e**

Compounds **7a**–**e** (0.5 mmol) and tetrachloro-*p*-benzoquinone (183 mg, 0.75 mmol) were dissolved in dichloroethane (7 mL), and the mixture was refluxed for 6 h. Then, dichloroethane was evaporated, and the residue was recrystallized from toluene to obtain **9a**–**e**.

2-(3,6-Diphenyl-1,2,4-triazin-5-yl)-1-hydroxy-3-methoxy-10-methylacridin-9(10*H*)-one **9a**. Yellow powder, m.p. > 250 °C. 54% yield. ^1^H NMR (400 MHz, DMSO-d_6_) δ 15.38 (s, 1H), 8.79–8.80 (br s, 1H), 8.48–8.58 (m, 1H), 8.18–8.27 (m, 2H), 8.00–8.13 (m, 1H), 7.78–7.91 (m, 2H), 7.56–7.70 (m, 3H), 7.30–7.45 (m, 4H), 6.63 (s, 1H), 3.93 (s, 3H), 3.73 (s, 3H). ^13^C NMR (101 MHz, DMSO-d_6_) δ 179.6, 161.7, 162.3, 161.1, 158.9, 152.4, 149.9, 145.1, 141.7, 137.2, 135.0, 134.5, 129.4, 128.0, 127.7, 125.5, 125.2, 123.7, 122.0, 119.9, 116.1, 105.2, 103.7, 87.6, 56.1, 34.4. Anal. Calcd for C_30_H_22_N_4_O_3_: C, 74.06; H, 4.56; N, 11.52%. Found: C, 75.31; H, 4.85; N, 11.38%.

1-Hydroxy-3-methoxy-10-methyl-2-(3-phenyl-6-(*p*-tolyl)-1,2,4-triazin-5-yl)acridin-9(10*H*)-one **9b**. Yellow powder, m.p. > 250 °C. 67% yield. ^1^H NMR (400 MHz, DMSO-d_6_) δ 15.39 (s, 1H), 8.47–8.49 (m, 2H), 8.26–8.28 (m, 1H), 7.84–7.90 (m, 2H), 7.59–7.62 (m, 3H), 7.48 (d, *J* = 7.8 Hz, 2H), 7.37–7.41 (m, 1H), 7.17 (d, *J* = 7.8 Hz, 2H), 6.64 (s, 1H), 3.94 (s, 3H), 3.75 (s, 3H), 2.25 (s, 3H). ^13^C NMR (101 MHz, DMSO-d_6_) δ 179.9, 162.5, 161.8, 161.1, 158.3, 153.0, 145.3, 142.0, 139.1, 134.9, 134.5, 132.4, 131.7, 129.1 (2C), 128.9 (2C), 127.8 (4C), 125.5, 122.4, 120.1, 116.5, 105.6, 104.0, 88.0, 56.4, 34.7, 20.8. Anal. Calcd for C_31_H_24_N_4_O_3_: C, 74.39; H, 4.83; N, 11.19%. Found: C, 74.29; H, 4.82; N, 11.07%. 

1-Hydroxy-3-methoxy-10-methyl-2-(6-phenyl-3-(pyridin-2-yl)-1,2,4-triazin-5-yl)acridin-9(10*H*)-one **9c**. Yellow powder, m.p. > 250 °C. 80% yield. ^1^H NMR (400 MHz, DMSO-d_6_) δ 15.37 (s, 1H), 8.84–8.85 (m, 1H), 8.52–8.54 (m, 1H), 8.22–8.24 (m, 1H), 8.05–8.09 (m, 1H), 7.82–7.87 (m, 2H), 7.60–7.64 (m, 3H), 7.35–7.39 (m, 4H), 6.63 (s, 1H), 3.93 (s, 3H), 3.73 (s, 3H). ^13^C NMR (100 MHz, DMSO-d_6_) δ 179.6, 162.2, 161.7, 161.1, 158.9, 153.2, 152.4, 149.9, 145.1, 141.8, 137.2, 135.0, 134.5, 129.4, 128.0 (2C), 127.7 (2C), 125.4, 125.2, 123.7, 122.0, 119.9, 116.1, 105.2, 103.7, 87.6, 56.0, 34.4. Anal. Calcd for: C_29_H_21_N_5_O_3_: C, 71.45; H, 4.34; N, 14.37%; Found: 71.25; H, 4.35; N, 14.73%.

1-Hydroxy-3-methoxy-2-(6-(4-methoxyphenyl)-3-(pyridin-2-yl)-1,2,4-triazin-5-yl)-10-methylacridin-9(10*H*)-one **9d**. Yellow powder, m.p. > 250 °C. 79% yield. ^1^H NMR (400 MHz, DMSO-d_6_) δ 15.27 (s, 1H), 8.81–8.82 (m, 1H), 8.55 (d, *J* = 7.9 Hz, 1H), 8.27 (d, *J* = 7.9 Hz, 1H), 7.99–8.03 (m, 1H), 7.81–7.85 (m, 2H), 7.53–7.58 (m, 3H), 7.33–7.37 (m, 1H), 6.87 (d, *J* = 8.5 Hz, 2H), 6.63 (s, 1H), 3.97 (s, 3H), 3.76 (s, 6H). ^13^C NMR (101 MHz, DMSO-d_6_) δ 179.7, 162.4, 162.0, 161.5, 160.1, 158.5, 153.1, 152.2, 150.0, 145.5, 142.4, 137.1, 135.3, 129.1 (2C), 128.4 (2C), 127.5, 127.0, 123.6, 122.2, 120.1, 116.3, 113.6 (2C), 104.9, 87.9, 56.1, 55.9, 34.3. Anal. Calcd for: C_30_H_23_N_5_O_4_: C, 69.62; H, 4.48; N, 13.53%; Found: C, 69.49; H, 4.39; N, 13.40%.

2-(3,6-bis(4-Methoxyphenyl)-1,2,4-triazin-5-yl)-1-hydroxy-3-methoxy-10-methylacridin-9(10*H*)-one **9e**. Yellow powder, m.p. > 250 °C. 76% yield. ^1^H NMR (400 MHz, DMF-d_7_) δ 15.51 (s, 1H), 8.53 (d, *J* = 8.8 Hz, 2H), 8.41 (d, *J* = 8.0 Hz, *J* = 1.7 Hz, 1H), 8.00 (d, *J* = 8.7 Hz, 1H), 7.94 (ddd, *J* = 8.7 Hz, *J* = 7.0 Hz, *J* = 1.7 Hz, 1H), 7.66 (d, *J* = 8.8 Hz, 2H), 7.47 (dd, *J* = 8.0 Hz, *J* = 7.0 Hz, 1H), 7.21 (d, *J* = 8.8 Hz, 2H), 6.96 (d, *J* = 8.8 Hz, 2H), 6.84 (s, 1H), 4.09 (s, 3H), 3.94 (s, 3H), 3.89 (s, 3H), 3.78 (s, 3H). ^13^C NMR (101 MHz, DMF-d_7_) δ 180.8, 163.4, 162.9, 162.5, 161.4, 160.9, 158.1, 153.2, 146.0, 142.7, 135.0, 129.8 (2C), 129.7 (2C), 128.5, 127.7, 126.0, 122.5, 121.0, 116.6, 114.7 (2C), 114.0 (2C), 106.8, 104.8, 88.2, 67.0, 56.4, 55.5, 55.2. Anal. Calcd for: C_32_H_26_N_4_O_5_: C, 70.32; H, 4.79; N, 10.25%. Found: C, 70.39; H, 4.86; N, 10.42%.

#### 3.1.3. Synthesis of Compounds **10a**–**d**

Compounds **9a**–**d** (0.2 mmol) were suspended in 1,2-dichlorobenzene (15 mL), 2,5-norbornadiene (0.16 mL, 1.6 mmol) was added, and the resulting mixture was stirred at 215 °C in an argon atmosphere for 20 h in the autoclave. The solvent was removed under reduced pressure, and the residue was purified by flash chromatography (using DCM and AcOEt (9:1) as eluents) to obtain pure **10a**–**d**.

2-(3,6-Diphenylpyridin-2-yl)-1-hydroxy-3-methoxy-10-methylacridin-9(10*H*)-one **10a**. Yellow powder, m.p. > 250 °C. 80% yield. ^1^H NMR (400 MHz, DMSO-d_6_) δ 14.88 (s, 1H), 8.30–8.37 (m, 1H), 8.07–8.13 (m, 2H), 7.94 (d, *J* = 8.0 Hz, 1H), 7.78–7.87 (m, 3H), 7.43–7.51 (m, 2H), 7.31–7.43 (m, 2H), 7.25–7.30 (m, 2H), 7.13–7.25 (m, 3H), 6.47 (s, 1H), 3.94 (s, 3H), 3.74 (s, 3H). ^13^C NMR (101 MHz, CDCl_3_) δ 180.5, 163.2, 162.5, 156.3, 151.5, 144.5, 141.9, 139.9, 139.7, 137.9, 127.7, 133.8, 128.6 (2C), 128.5, 127.7, 127.2, 126.9, 126.4, 121.5, 121.0, 119.5, 114.7, 110.8, 105.1, 86.1, 55.4, 34.1. Anal. Calcd for: C_32_H_24_N_2_O_3_: C, 79.32; H, 4.99; N, 5.78%. Found: C, 79.28; H, 4.95; N, 5.75%.

1-Hydroxy-3-methoxy-10-methyl-2-(6-phenyl-3-(*p*-tolyl)pyridin-2-yl)acridin-9(10*H*)-one **10b**. Yellow powder, m.p. > 250 °C. 83% yield. ^1^H NMR (400 MHz, DMSO-d_6_) δ 14.87 (s, 1H), 8.31–8.37 (m, 1H), 8.06–8.11 (m, 2H), 7.93 (d, *J* = 8.0 Hz, 1H), 7.78–7.84 (m, 3H), 7.43–7.50 (m, 2H), 7.32–7.43 (m, 2H), 7.13–7.19 (m, 2H), 6.99–7.04 (m, 2H), 6.50 (s, 1H), 3.96 (s, 3H), 3.77 (s, 3H), 2.25 (s, 3H). ^13^C NMR (101 MHz, CDCl_3_) δ 180.4, 163.3, 162.4, 156.1, 151.5, 144.4, 141.9, 139.8, 137.9, 137.7, 137.0, 136.5, 133.8, 128.6, 128.5, 128.4, 128.3, 127.2, 126.4, 121.5, 120.9, 119.4, 114.7, 110.9, 105.1, 86.2, 67.9, 55.5, 34.1. Anal. Calcd for: C_33_H_26_N_2_O_3_: C, 79.50; H, 5.26; N, 5.62%. Found: C, 79.45; H, 5.21; N, 5.58%.

Crystal data for **10b**: C_33_H_26_N_2_O_3_ (M = 498.59), monoclinic, space group P1_21_/c_1_ at 295(2) K, a = 14.2820(4), b = 8.46146(13) and c = 26.2682(6) Å, α = 90°, β = 97.089(3), γ = 90°, V = 3150.15(12) Å^3^, *Z* = 4, d_calc_ = 1.051 g/cm^–3^, *μ*(MoKα) = 0.068 mm^–1^, F(000) = 1048. A total of 233,592 reflections were collected (8308 independent reflections, R_int_ = 0.1300) and used in the refinement, which converged to *wR*_2_ = 0.1412, GOOF 0.955 for all independent reflections [*R*_1_ = 0.1332 was calculated for 8308 reflections with *I* > 2σ(*I*)]. CCDC 2,231,907 contains the crystallographic data for this compound.

1-Hydroxy-3-methoxy-10-methyl-2-(5-phenyl-[2,2’-bipyridin]-6-yl)acridin-9(10*H*)-one **10c**. Yellow powder, m.p. > 250 °C. 85% yield. ^1^H NMR (400 MHz, DMSO-d_6_) δ 14.91 (s, 1H), 8.65–8.71 (m, 1H), 8.47 (d, *J* = 8.8 Hz, 1H), 8.39 (d, *J* = 8.0 Hz, 1H), 8.34 (d, *J* = 8.0 Hz, 1H), 7.79–7.93 (m, 4H), 7.32–7.43 (m, 2H), 7.25–7.32 (m, 2H), 7.15–7.25 (m, 3H), 6.49 (s, 1H), 3.95 (s, 3H), 3.73 (s, 3H). ^13^C NMR (101 MHz, DMSO-d_6_) δ 180.3, 163.8, 161.9, 155.9, 154.6, 151.4, 149.8, 144.9, 142.5, 139.8, 139.3, 138.5, 137.6, 134.9, 128.5, 128.3, 127.7, 126.1, 124.4, 122.3, 121.2, 120.7, 119.8, 116.6, 110.8, 104.7, 87.9, 56.4, 34.9. Anal. Calcd for: C_31_H_23_N_3_O_3_: C, 76.69; H, 4.77; N, 8.65%. Found: C, 76.61; H, 4.72; N, 8.64%.

1-Hydroxy-3-methoxy-2-(5-(4-methoxyphenyl)-[2,2’-bipyridin]-6-yl)-10-methylacridin-9(10*H*)-one **10d**. Yellow powder, m.p. > 250 °C. 75% yield. ^1^H NMR (400 MHz, DMSO-d_6_) δ 14.89 (s, 1H), 8.64–8.69 (m, 1H), 8.44 (d, *J* = 8.0 Hz, 1H), 8.32–8.41 (m, 2H), 7.78–7.90 (m, 4H), 7.31–7.40 (m, 2H), 6.18–6.24 (m, 2H), 6.72–6.80 (m, 2H), 6.51 (s, 1H), 3.96 (s, 3H),3.76 (s, 3H), 3.71 (s, 3H). ^13^C NMR (126 MHz, DMF-d_7_) δ 181.1, 164.5, 159.7, 156.6, 154.8, 152.1, 150.0, 145.5, 143.1, 139.7, 138.8, 137.6, 135.0, 132.7, 130.2 (2C), 126.4, 124.5, 122.4, 121.3, 121.2, 119.9, 116.7, 113.9, 111.7, 105.3, 88.0, 56.4, 55.4, 31.4. Anal. Calcd for: C_32_H_25_N_3_O_4_: C, 74.55; H, 4.89; N, 8.15%. Found: C, 74.50; H, 4.84; N, 8.09%.

#### 3.1.4. Synthesis of Compounds **12a** and **12b**

In acetic acid (10 mL), 1,3-Dihydroxy-9*H*-xanthen-9-one **11** (228 mg, 1.0 mmol), 1,2,4-triazine **6a**,**b** (1.1 mmol), and methanesulfonic acid (288 mg, 3.0 mmol) were dissolved and the mixture allowed to stand for 48 h. The mixture was poured in water and quenched with an NaHCO_3_ solution, and the precipitate was filtered and recrystallized from ethyl acetate yielding pure **12a**,**b**.

4-(3,6-Diphenyl-2,5-dihydro-1,2,4-triazin-5-yl)-1,3-dihydroxy-9*H*-xanthen-9-one **12a**. White powder, m.p. > 250 °C. 64% yield. ^1^H NMR (400 MHz, DMSO-d_6_) δ 12.95 (br s, 1H), 8.60–12.40 (br s, 2H), 8.00–8.02 (m, 1H), 7.92–7.94 (m, 2H), 7.87–7.89 (m, 2H), 7.75–7.79 (m, 1H), 7.68–7.70 (m, 1H), 7.38–7.46 (m, 3H), 7.25–7.37 (m, 4H), 6.62 (s, 1H), 6.18 (s, 1H). ^13^C NMR (101 MHz, DMSO-d_6_) δ 177.8, 169.2, 161.7, 157.0, 155.1, 149.1, 141.5, 135.7, 134.7, 132.8, 130.4, 128.9, 128.3 (2C), 128.2 (2C), 126.2 (2C), 125.1 (2C), 124.9, 123.9, 119.9, 117.4, 110.4, 100.2, 98.9, 45.0. Anal. Calcd for: C_28_H_19_N_3_O_4_: C, 72.88; H, 4.15; N, 9.11%. Found: C, 73.02; H, 4.12; N, 9.30%.

1,3-Dihydroxy-4-(3-(methylthio)-2,5-dihydro-1,2,4-triazin-5-yl)-9*H*-xanthen-9-one **12b**. Off-white powder, m.p. = 220–230 °C dec. 59% yield. ^1^H NMR (400 MHz, DMSO-d_6_) δ 13.24 (br s, 1H), 12.99 (br s, 1H), 12.38 (br s, 1H), 8.15 (d, *J* = 7.7 Hz, 1H), 7.91 (dd, *J* = 7.7 Hz, *J* = 7.5 Hz, 1H), 7.64 (d, *J* = 8.0 Hz, 1H), 7.51 (dd, *J* = 7.5 Hz, *J* = 8.0 Hz, 1H), 7.35 (d, *J* = 2.9 Hz, 1H), 6.60 (s, 1H), 5.92 (s, 1H), 2.66 (s, 3H). ^13^C NMR (101 MHz, DMSO-d_6_) δ 179.8, 164.5, 163.1, 160.2, 156.1, 155.0, 144.0, 136.0, 125.3, 125.0, 119.6, 117.6, 102.5, 102.1, 98.0, 43.2, 13.4. Anal. Calcd for: C_17_H_13_N_3_O_4_S: C, 57.46; H, 3.69; N, 11.82%. Found: C, 57.59; H, 3.79; N, 11.96%.

#### 3.1.5. Synthesis of Compounds **14a** and **14b**

In acetic acid (10 mL), 1,3-Dimethoxy-9*H*-xanthen-9-one **13** (1.0 mmol), 1,2,4-triazine **6a** (233 mg, 1.1 mmol), and methanesulfonic acid (288 mg, 3.0 mmol) were dissolved and the mixture allowed to stand for 48 h. The mixture was poured in water and quenched with NaHCO_3_ solution, and the precipitate was filtered and recrystallized from ethyl acetate yielding pure **14a**–**b**.

4-(3,6-Diphenyl-2,5-dihydro-1,2,4-triazin-5-yl)-1,3-dimethoxy-9*H*-xanthen-9-one **14a**. Off-white powder, m.p. > 250 °C. 72% yield. ^1^H NMR (600 MHz, DMSO-d_6_) δ 11.30 (s, 1H), 8.01–8.00 (m, 1H), 7.85–7.83 (m, 2H), 7.79–7.76 (m, 1H), 7.72–7.70 (m, 1H), 7.68–7.67 (m, 2H), 7.44–7.35 (m, 4H), 7.28–7.23 (m, 3H), 6.68 (s, 1H), 6.58 (s, 1H), 4.11 (s, 3H), 3.93 (s, 3H). ^13^C NMR (151 MHz, DMSO-d_6_) δ 173.6, 161.6, 161.2, 157.0, 154.3, 149.1, 140.0, 135.9, 134.4, 133.3, 130.3, 128.8, 128.3 (4C), 126.0 (2C), 125.8, 124.9 (2C), 124.1, 122.0, 117.3, 111.3, 106.0, 91.8, 56.7, 56.2, 46.0. Anal. Calcd for: C_30_H_23_N_3_O_4_: C, 73.61; H, 4.74; N, 8.58%. Found: C, 73.70; H, 4.87; N, 8.39%.

4-(3,6-Diphenyl-2,5-dihydro-1,2,4-triazin-5-yl)-1,3,6-trimethoxy-9*H*-xanthen-9-one **14b**. Off-white powder, m.p. > 250 °C. 68% yield. ^1^H NMR (400 MHz, DMSO-d_6_) δ 11.33 (s, 1H), 7.90–7.92 (m, 1H), 7.85–7.86 (m, 2H), 7.68–7.69 (m, 2H), 7.38–7.44 (m, 3H), 7.24–7.29 (m, 3H), 7.14 (s, 1H), 6.93–6.95 (m, 1H), 6.65 (s, 1H), 6.58 (s, 1H), 4.09 (s, 3H), 3.91 (s, 6H). ^13^C NMR (100 MHz, DMSO-d_6_) δ 172.8, 164.0, 161.2, 161.0, 157.0, 155.8, 149.9 (2C), 140.0, 135.8, 133.2, 130.1, 128.6, 128.1 (5C), 127.2, 125.9 (2C), 124.8 (2C), 115.8, 112.5, 111.3, 105.9, 100.1, 91.9, 56.5, 56.1, 56.0, 46.0. Anal. Calcd for: C_31_H_25_N_3_O_5_: C, 71.67; H, 4.85; N, 8.09%. Found: C, 71.56; H, 5.02; N, 7.95%.

#### 3.1.6. Synthesis of Compounds **15a** and **15b**

Compound **14a**,**b** (0.5 mmol) and tetrachloro-*p*-benzoquinone (183 mg, 0.75 mmol) were dissolved in dichloroethane, and the mixture was refluxed for 6 h. Then dichloroethane was evaporated, and the residue was recrystallized from toluene to obtain **15a**,**b**.

4-(3,6-Diphenyl-1,2,4-triazin-5-yl)-1,3-dimethoxy-9*H*-xanthen-9-one **15a**. Light yellow powder, m.p. = 230–233 °C. 81% yield. ^1^H NMR (600 MHz, CDCl_3_) δ 8.65–8.63 (m, 2H), 8.25–8.24 (m, 1H), 7.56–7.54 (m, 6H), 7.32–7.24 (m, 4H), 7.10–7.08 (m, 1H), 6.33 (s, 1H), 4.06 (s, 3H), 3.65 (s, 3H). ^13^C NMR (151 MHz, CDCl_3_) δ 175.5, 164.0, 162.2, 162.0, 158.4, 156.6, 154.7, 152.3, 135.6, 135.1, 134.2, 131.6, 129.6, 129.0 (2C), 128.5 (2C), 128.4 (2C), 128.1 (2C), 126.7, 124.3, 122.8, 117.1, 107.3, 106.7, 90.7, 56.7, 56.1. Anal. Calcd for: C_30_H_21_N_3_O_4_: C, 73.91; H, 4.34; N, 8.62%. Found: C, 73.83; H, 4.54; N, 8.79%.

4-(3,6-Diphenyl-1,2,4-triazin-5-yl)-1,3,6-trimethoxy-9*H*-xanthen-9-one **15b**. Light yellow powder, m.p. = 248–249 °C. 81% yield. ^1^H NMR (400 MHz, DMSO-d_6_) δ 8.51–8.53 (m, 2H), 7.90 (d, *J* = 8.9 Hz, 1H), 7.63–7.65 (m, 3H), 7.53–7.55 (m, 2H), 7.32–7.34 (m, 3H), 6.91 (dd, *J* = 8.9 Hz, *J* = 2.3 Hz, 1H), 6.74 (d, *J* = 2.3 Hz, 1H), 6.70 (s, 1H), 3.99 (s, 3H), 3.79 (s, 3H), 3.78 (s, 3H). ^13^C NMR (101 MHz, DMSO-d_6_) δ 172.7, 164.0, 163.2, 161.7, 161.4, 158.4, 155.7, 155.3, 152.2, 135.0, 134.4, 131.8, 129.5, 129.2 (2C), 128.2 (2C), 127.9 (2C), 127.8 (2C), 127.1, 115.7, 113.4, 105.7, 105.3, 99.8, 92.2, 59.7, 56.6, 56.1. Anal. Calcd for: C_31_H_23_N_3_O_5_: C, 71.94; H, 4.48; N, 8.12%. Found: C, 71.98; H, 4.54; N, 8.35%.

For images of ^1^H and ^13^C spectra of compounds **7**, **9**, **10**, **12**, **14**, and **15** see Supplementary Materials file.

### 3.2. Anticancer Activity Evaluating

#### 3.2.1. Cell Culture

The studies were carried out on cultured cells of human glioblastoma (A-172, ATCC CRL 1620) [29], human breast cancer (Hs578T, ATCC HTB-126) [30] and human embryonic kidney 293 cells (HEK-293, ATCC CRL 1573) [31] obtained from the shared research facility “Vertebrate cell culture collection” (Institute of Cytology, RAS, Russia). The cells were cultured in DMEM/F-12 medium containing 10% fetal bovine serum at 37 °C, 5% CO_2_, and 98% humidity. Subculturing using a 0.25% trypsin solution was performed when the culture reached ≥ 90% confluency. 

#### 3.2.2. Viability Assessment

The compounds were dissolved in DMSO. The solutions were diluted with DMEM/F-12 culture medium with 10% fetal bovine serum to the studied concentrations: 1, 2, 4, 8, 16, 32, 64, 128, 256 µM. In all cases, the concentration of DMSO in the final solution did not exceed 1%.

Cells were seeded in 96-well plates at a concentration of 4 × 10^3^ cells per well. After 24 h, test compounds were added to the wells in a given concentration range. Then the cells were incubated for 24 h, after which a solution of MTT (3-(4,5-dimethyl-2-thiazolyl)-2,5-diphenyl-2H-tetrazolium bromide) was added to the cultures at 20 µL (5 mg/mL) to the well. After 2 h, the medium was removed from the wells, and 200 µL of a 1:1 mixture of DMSO and isopropanol was added. The optical density was measured on a plate spectrophotometer at a wavelength of 570 nm.

#### 3.2.3. Statistical Analysis

Statistical data processing was carried out in the RStudio program (Version 1.4.1106 © 2009–2021 RStudio, PBC) using the R package (version 4.1.2). The cytotoxicity index (IC_50_) was calculated by plotting dose-response curves using the “drc” package [32].

Stock solutions of the tested compounds of the concentration 0.0125 M were prepared by diluting the suspension in an appropriate volume of DMSO, and then diluting the obtained solution in the nutrient medium DMEM/F-12 (10% FBS) to obtain solutions of the tested concentration in the range from 1 to 128 μM.

#### 3.2.4. Apoptosis

The cells of the lines A-172 and HEK-293 were incubated in 35 mm Petri dishes one day before adding the studied compounds and incubated under the conditions of 5% CO_2_, t = 37 °C, and 95% humidity. Then, solutions of the test compounds and cisplatin were added at a concentration of 6 μM. The cultures were returned to the incubator for 24 h, then the cells were stripped with 0.25% trypsin solution and centrifuged at 200× *g* for 5 min. The cell precipitate was resuspended in a 25 μL phosphate buffer. To the resulting suspension, 25 µL of freshly prepared dye mixture was added, pipetted, and incubated for 10 min in the dark. After that, 10 μL of the mixture was added to a cell counter slide (C100, RWD) for automatic counting.

#### 3.2.5. DNA Synthesis

Cells were pre-dispersed into the wells of a 96-well plate at a seeding concentration of 4 × 10^3^ cells per well the day before the addition of the test substances and incubated in a CO_2_ incubator for 24 h. Subsequently, suspensions of the test compounds were added at a concentration of 6 μM. After 24 h, EdU reagent was added, and the cultures were incubated for 2 h. Then the cells were fixed with formaldehyde and permeabilized with Triton X-100. The cells were then incubated for 30 min at room temperature with the reaction mixture, washed, stained with DAPI, and microscopically examined (Optika, Italy).

## 4. Conclusions

Herein, we report the synthesis of acridone-triazine, acridone-pyridine, and xanthone-triazine conjugates. The synthetic strategy involves the coupling reaction between hydroxy- and/or alkoxy derivatives of dibenzo-pyrones **11** and **13** or pyridones **2** with 1,2,4-triazines **6a**–**f**, followed by optional aromatization of the dihydrotriazine ring leading to aromatic 1,2,4-triazines and the Boger reaction of the triazines yielding pyridines. The antitumor activity was evaluated in vitro on several cell lines. The conjugates of acridone with 1,2,4-triazine, compounds **7a** and **7c**, were the most active among the compounds tested. These compounds show high activity on colorectal cancer (HCT116), glioblastoma (A-172), and breast ductal carcinoma (Hs578T) cell lines, combined with low toxicity on normal human embryonic kidney (HEK-293) cells, and can be used as a starting point in the development of new anticancer agents. It should be noted that aromatization of the dihydrotriazine ring appears to have little effect on activity but significantly reduces the solubility of the compounds. Pyridine derivatives of acridones demonstrated no anticancer activity on the tested cell lines. Conjugates of xanthones with 1,2,4-triazines showed moderate activity on colorectal cancer HCT116 cells. Preliminary mechanistic studies of anticancer activity using the Annexin V assay demonstrated that compound **7e** activates apoptotic mechanisms and inhibits proliferation in glioblastoma cells.

## Figures and Tables

**Table 1:** Cytotoxicity index (IC_50_) on colorectal cancer (HCT116), glioblastoma (A-172), breast ductal carcinoma (Hs578T), and normal human embryonic kidney (HEK-293) cells for compounds **2**, **7a**–**e**, **9e**, **12a**–**b**, **14a**–**b**, and **15b**, μM.

Compound	HCT116	A-172	Hs-578T	HEK-293
**2**	43.45 ± 8.54			
**7a**	14.56 ± 3.81	6.92 ± 2.29	7.13 ± 2.83	115.15 ± 15.56
**7b**	>100			
**7c**	1.37 ± 0.48			
**7d**	>100			
**7e**	6.1 ± 1.74	3.56 ± 0.36	1.74 ± 0.08	47.43 ± 3.99
**9e**	7.15 ± 1.02			
**10a**		>128	>128	>128
**10c**		>128	>128	>128
**12a**	>100			
**12b**	>100			
**14a**	28.01 ± 5.64			
**14b**	17.99 ± 1.63			
**15b**	>100			
Etoposide	1.08 ± 0.34			
Cisplatin		39.5 ± 6.4	122.0 ± 13.0	59.9 ± 20.0

## Data Availability

Data is contained within the article and Supplementary Materials.

## Supplementary Materials

The following supporting information can be downloaded at: https://www.mdpi.com/article/10.3390/ph16030403/s1.
