# Supplementary material for: Xanthone-1,2,4-triazine and Acridone-1,2,4-triazine Conjugates: Synthesis and Anticancer Activity
Source: Pharmaceuticals (Basel). 2023 Mar 7;16(3):403. doi: 10.3390/ph16030403 (PMC10058176; doi:10.3390/ph16030403)
Supplement: Supplementary file 1 [file pharmaceuticals-16-00403-s001.zip › pharmaceuticals-2153485-supplementary.pdf]

<u>Supporting Information</u>

Xanthone-1,2,4-triazine and Acridone-1,2,4-triazine Conjugates: Synthesis and Anticancer Activity

Sougata Santra[1], Ainur D. Sharapov[1], Ramil F. Fatykhov[1], Anastasya P. Potapova[1], Igor A. Khalymbadzha[1], Maria I. Valieva[1], Dmitry S. Kopchuk[1], Grigory V. Zyryanov[1], Alexander S. Bunev[2], Vsevolod V. Melekhin[1,3], Vasiliy S. Gaviko[1,4], Andrey A. Zonov[1], and Oleg N. Chupakhin[1]

[1] Department of Organic and Biomolecular Chemistry, Ural Federal University, Mira 19, 620002, Ekaterinburg, Russian Federation;

[2] Medicinal Chemistry Center, Togliatti State University, Belorusskaya 14, 445020, Togliatti, Russian Federation;

[3] Ural State Medical University, Repina 3, 620028, Ekaterinburg, Russian Federation;

[4] M.N. Mikheev Institute of Metal Physics, Ural Branch of the Russian Academy of Sciences, Kovalevskoy Street 18, 620108, Ekaterinburg, Russian Federation

Table of Contents

Figure S1. ^{1}H NMR spectrum of **7a** 3
Figure S2. ^{13}C NMR spectrum of **7a** 3
Figure S3. ^{1}H NMR spectrum of **7b** 4
Figure S4. ^{13}C NMR spectrum of **7b** 4
Figure S5. ^{1}H NMR spectrum of **7c** 5
Figure S6. ^{13}C NMR spectrum of **7c** 5
Figure S7. ^{1}H NMR spectrum of **7d** 6
Figure S8. ^{13}C NMR spectrum of **7d** 6
Figure S9. ^{1}H NMR spectrum of **7e** 7
Figure S10. ^{13}C NMR spectrum of **7e** 7
Figure S11. ^{1}H NMR spectrum of **7f** 8
Figure S12. ^{13}C NMR spectrum of **7f** 8
Figure S13. ^{1}H NMR spectrum of **9b** 9
Figure S14. ^{13}C NMR spectrum of **9b** 9
Figure S15. ^{1}H NMR spectrum of **9c** 10
Figure S16. ^{13}C NMR spectrum of **9c** 10
Figure S17. ^{1}H NMR spectrum of **9e** 11
Figure S18. ^{13}C NMR spectrum of **9e** 11
Figure S19. ^{1}H NMR spectrum of **12a** 12
Figure S20. ^{13}C NMR spectrum of **12a** 12
Figure S21. ^{1}H NMR spectrum of **12b** 13
Figure S22. ^{13}C NMR spectrum of **12b** 13

Figure S23. ^{1}H NMR spectrum of **14a** 14
Figure S24. ^{13}C NMR spectrum of **14a** 14
Figure S25. ^{1}H NMR spectrum of **14b** 15
Figure S26. ^{13}C NMR spectrum of **14b** 15
Figure S27. ^{1}H NMR spectrum of **15a** 16
Figure S28. ^{13}C NMR spectrum of **15a** 16
Figure S29. HCT116 cell survival curve for compounds **2**, **7d**, **7f**, **9e**, **12a** and **14a** 17
Figure S30. HCT116 cell survival curve for compounds **7a**-**c**,**e**, **12b**, **14b**, **15b** 17

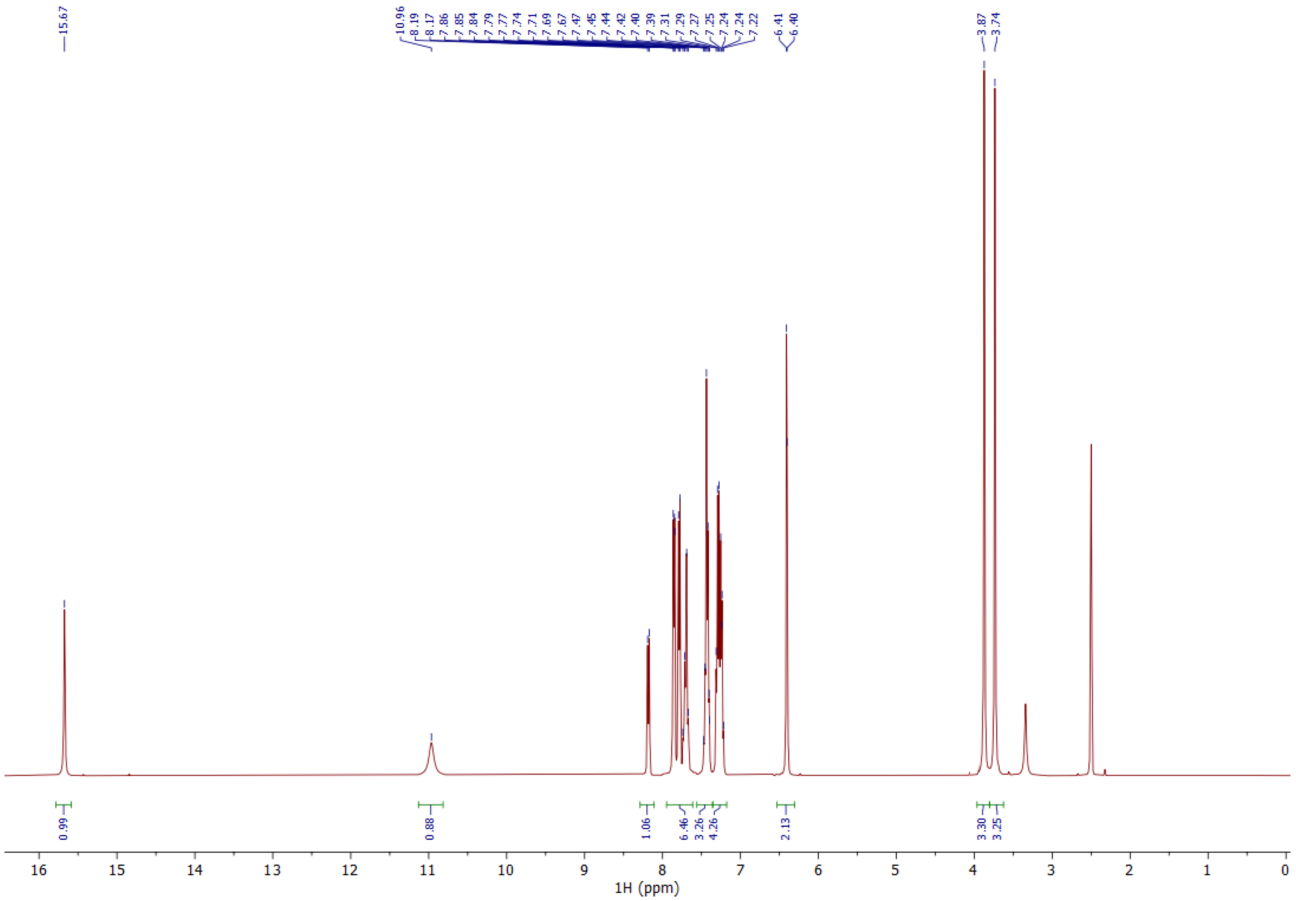

Figure S1. **^{1}H NMR spectrum of 7a**

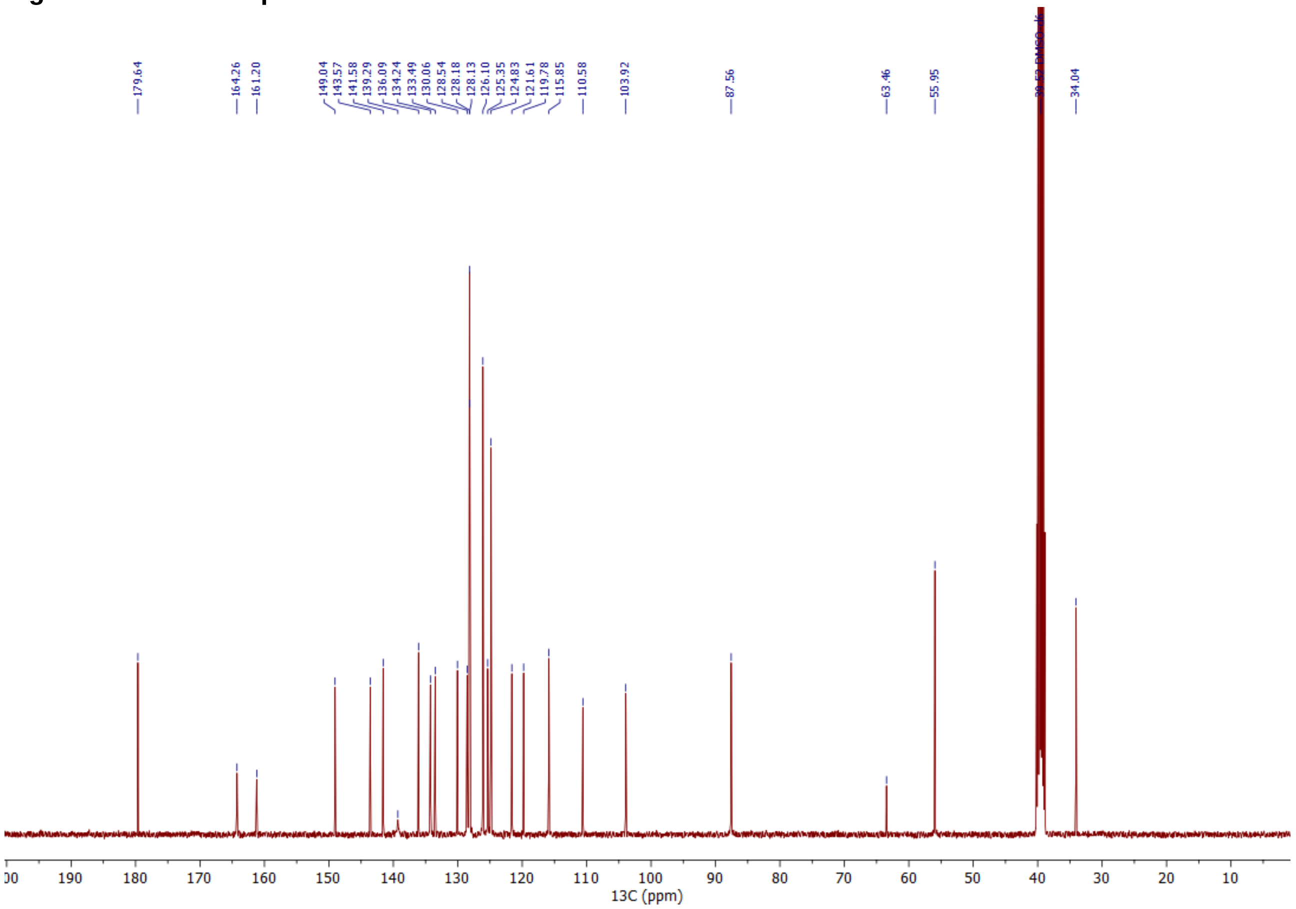

Figure S2. **^{13}C NMR spectrum of 7a**

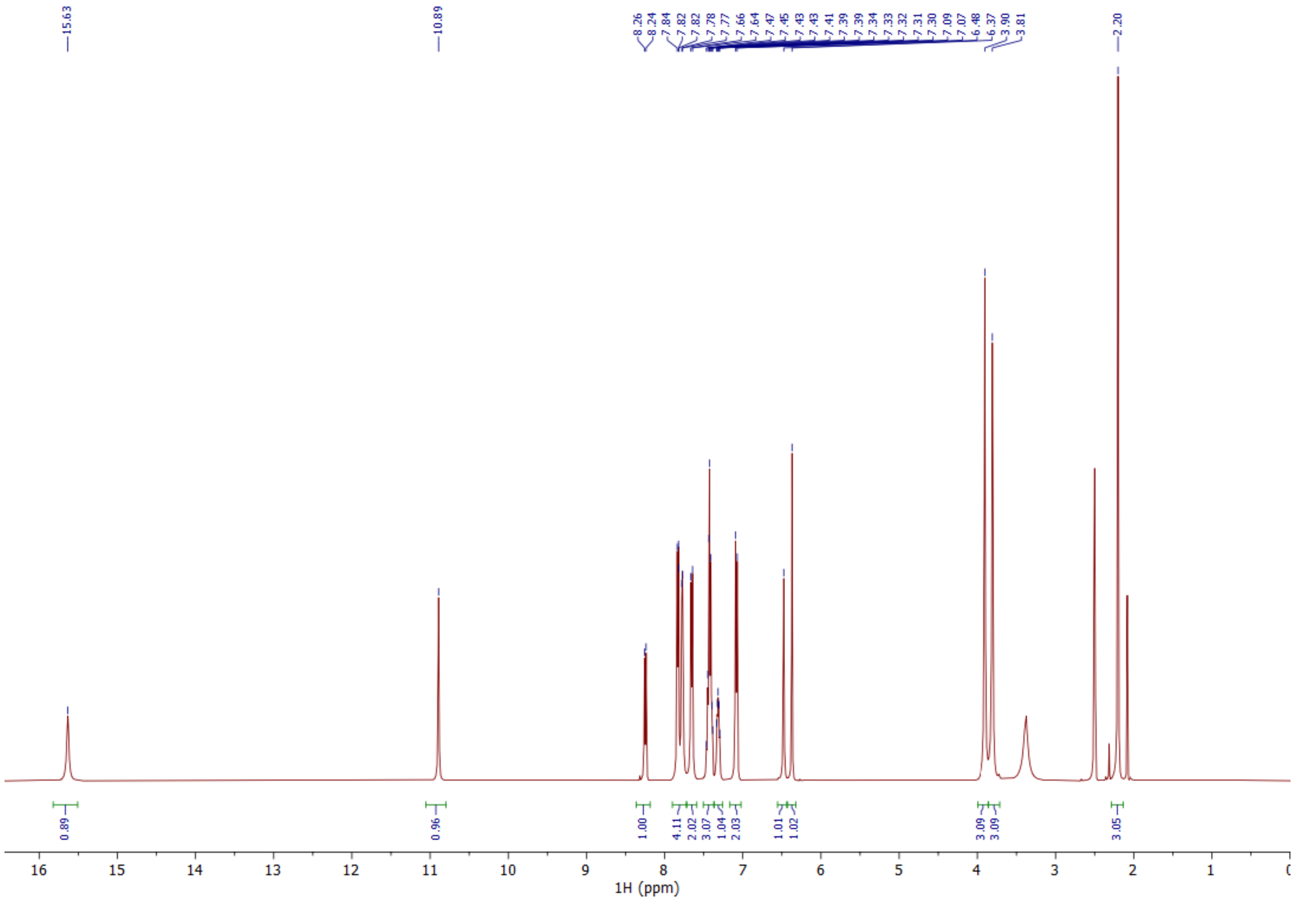

Figure S3. **^{1}H NMR spectrum of 7b**

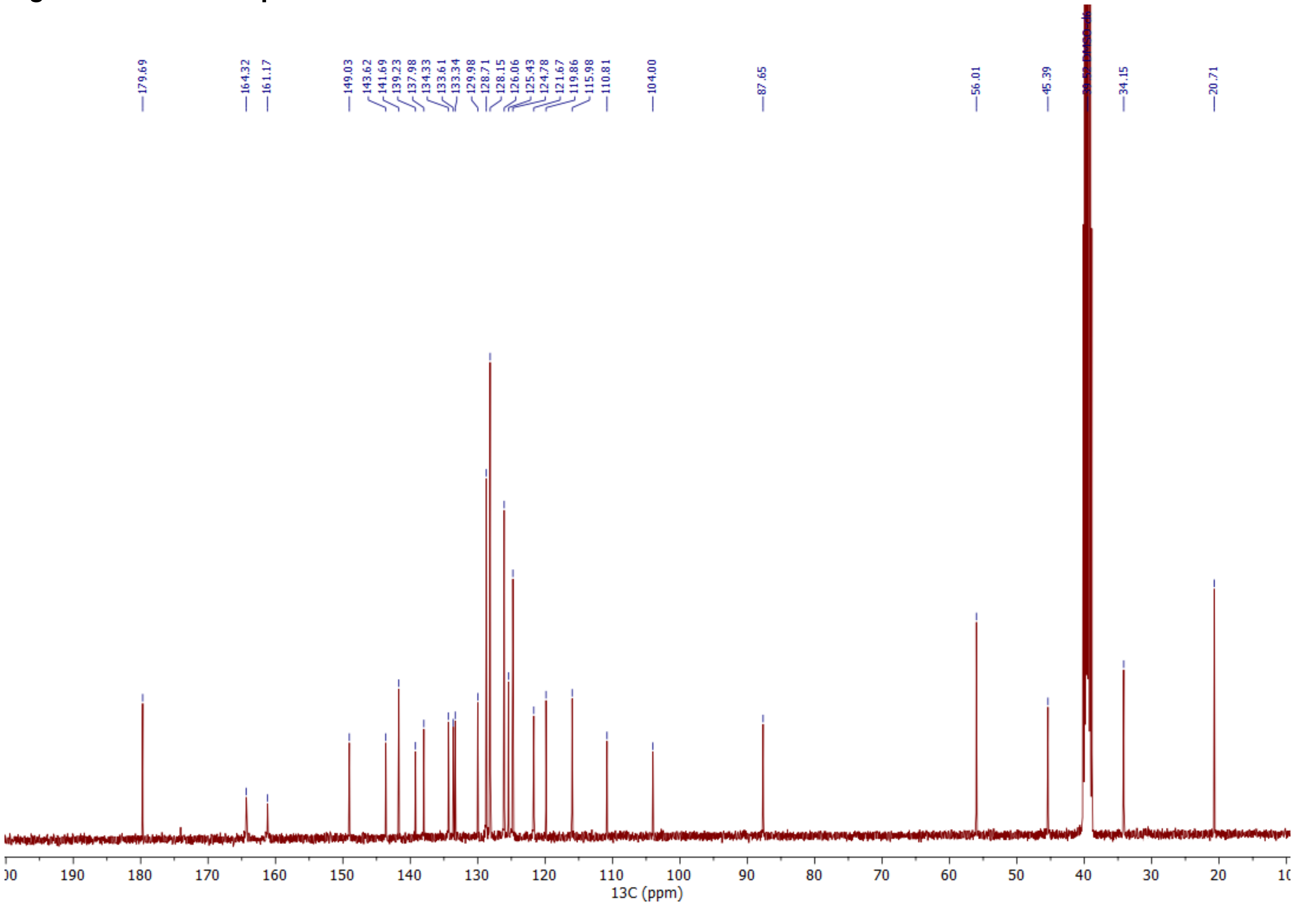

Figure S4. **^{13}C NMR spectrum of 7b**

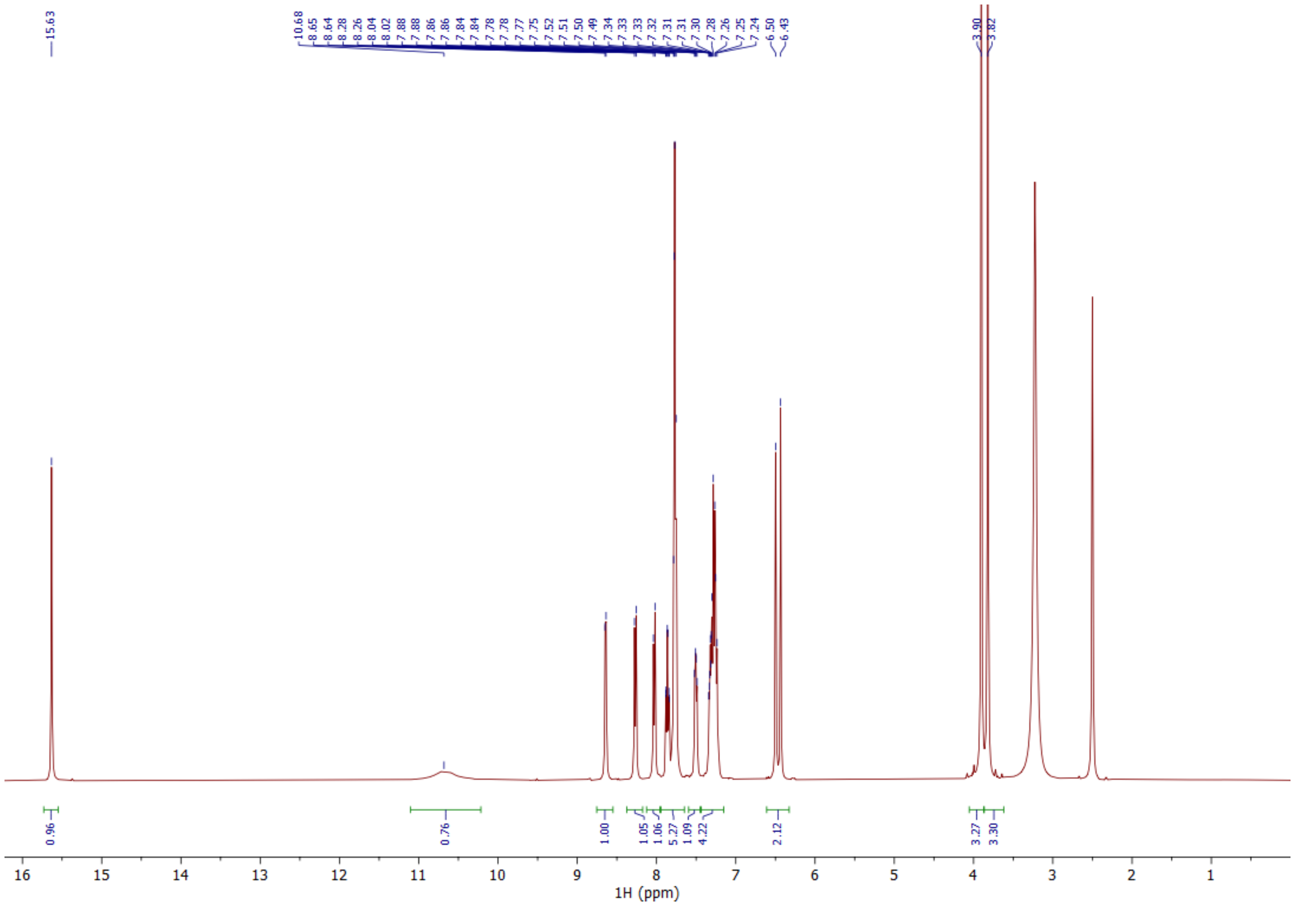

Figure S5. [1]**H NMR spectrum of 7c**

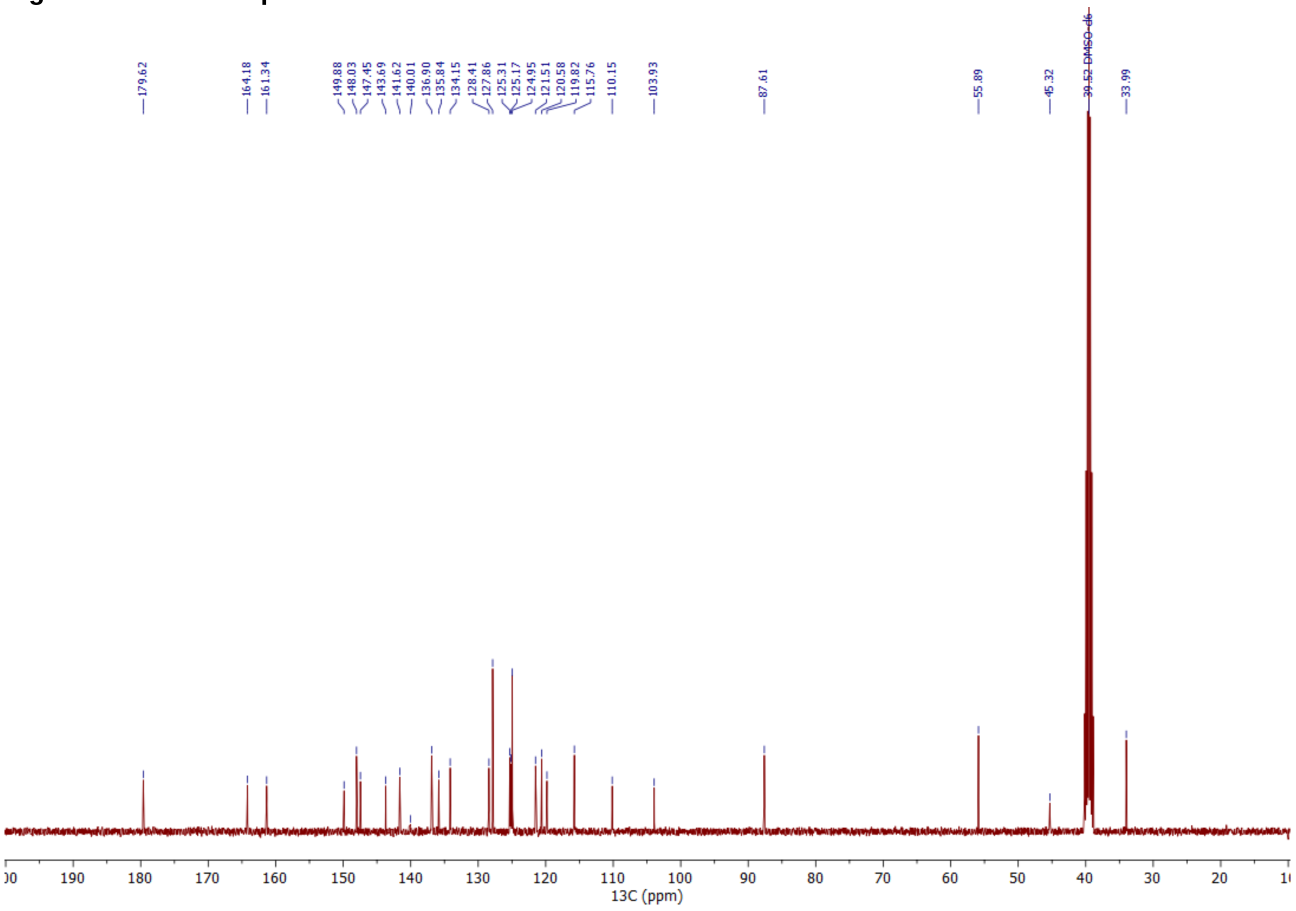

Figure S6. [13]**C NMR spectrum of 7c**

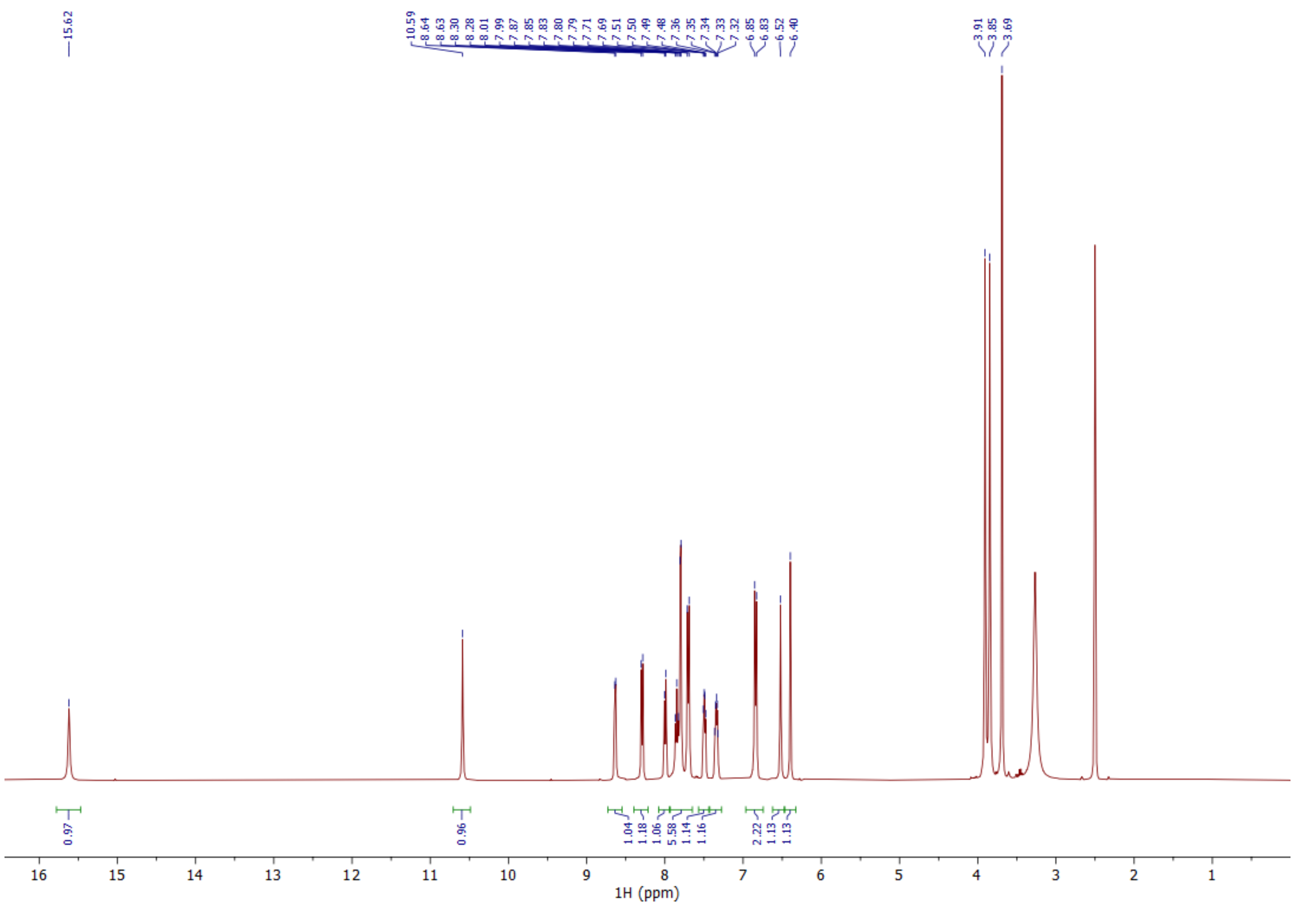

Figure S7. ^{1}**H NMR spectrum of 7d**

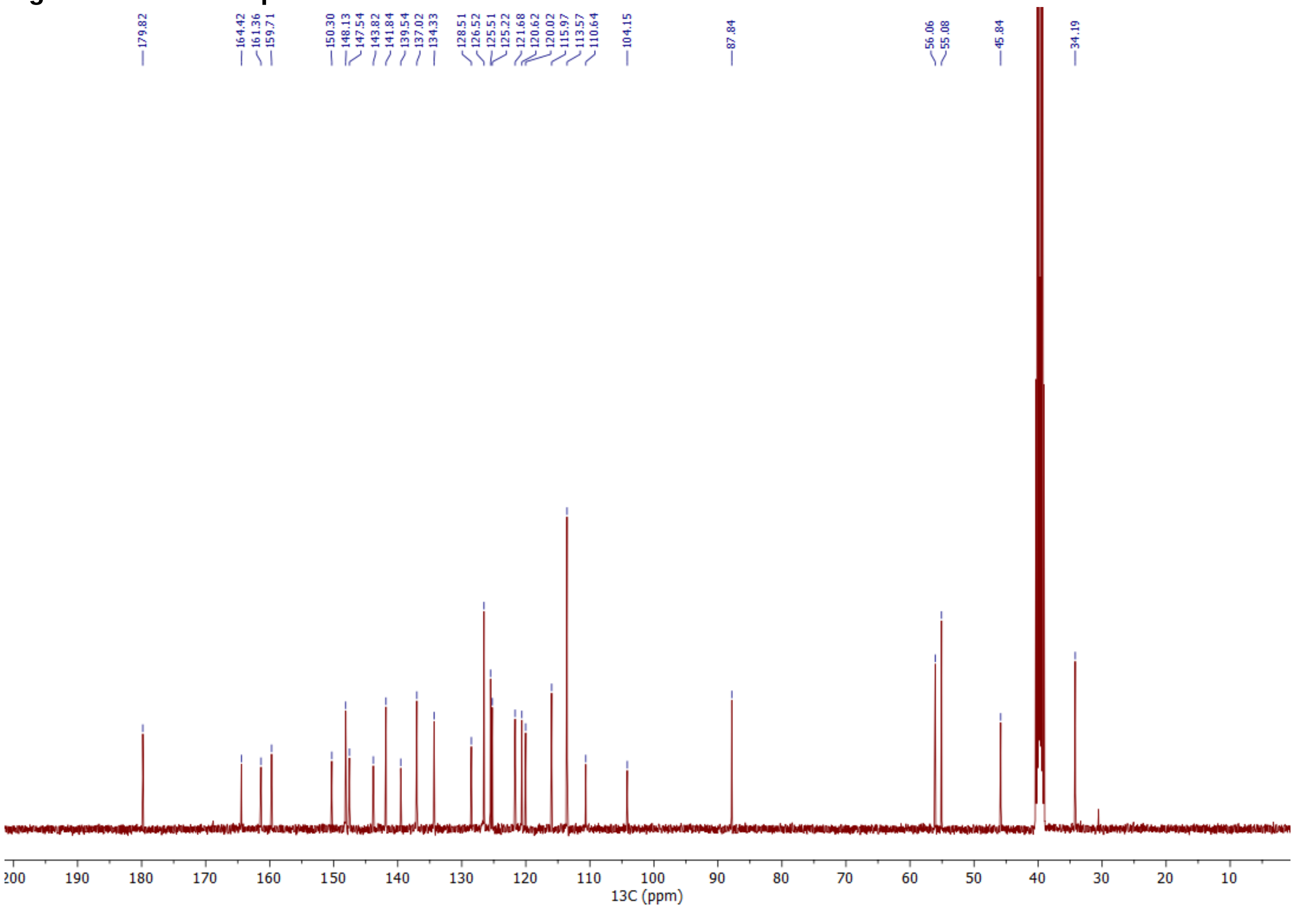

Figure S8. ^{13}**C NMR spectrum of 7d**

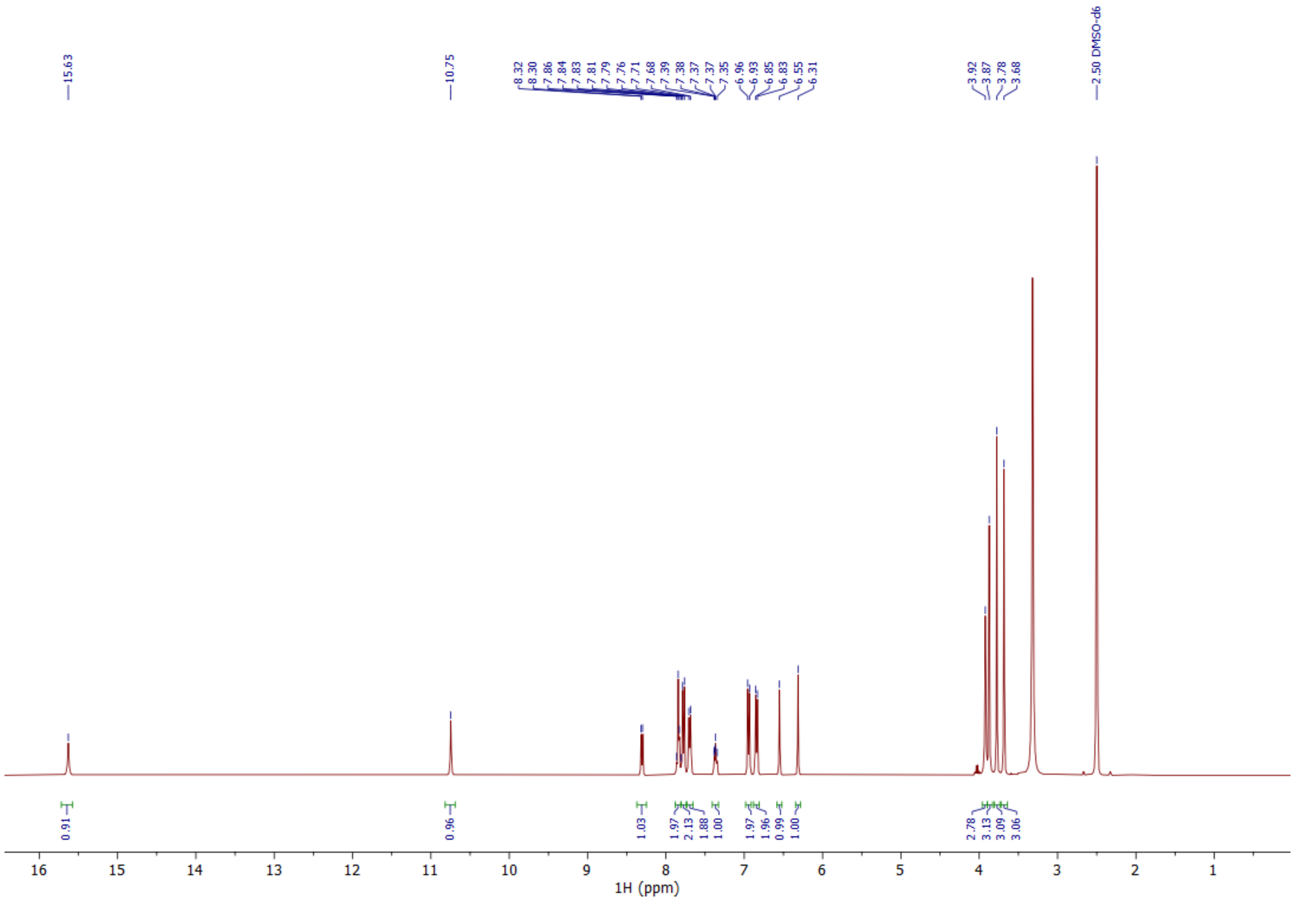

Figure S9. ^{1}H NMR spectrum of 7e

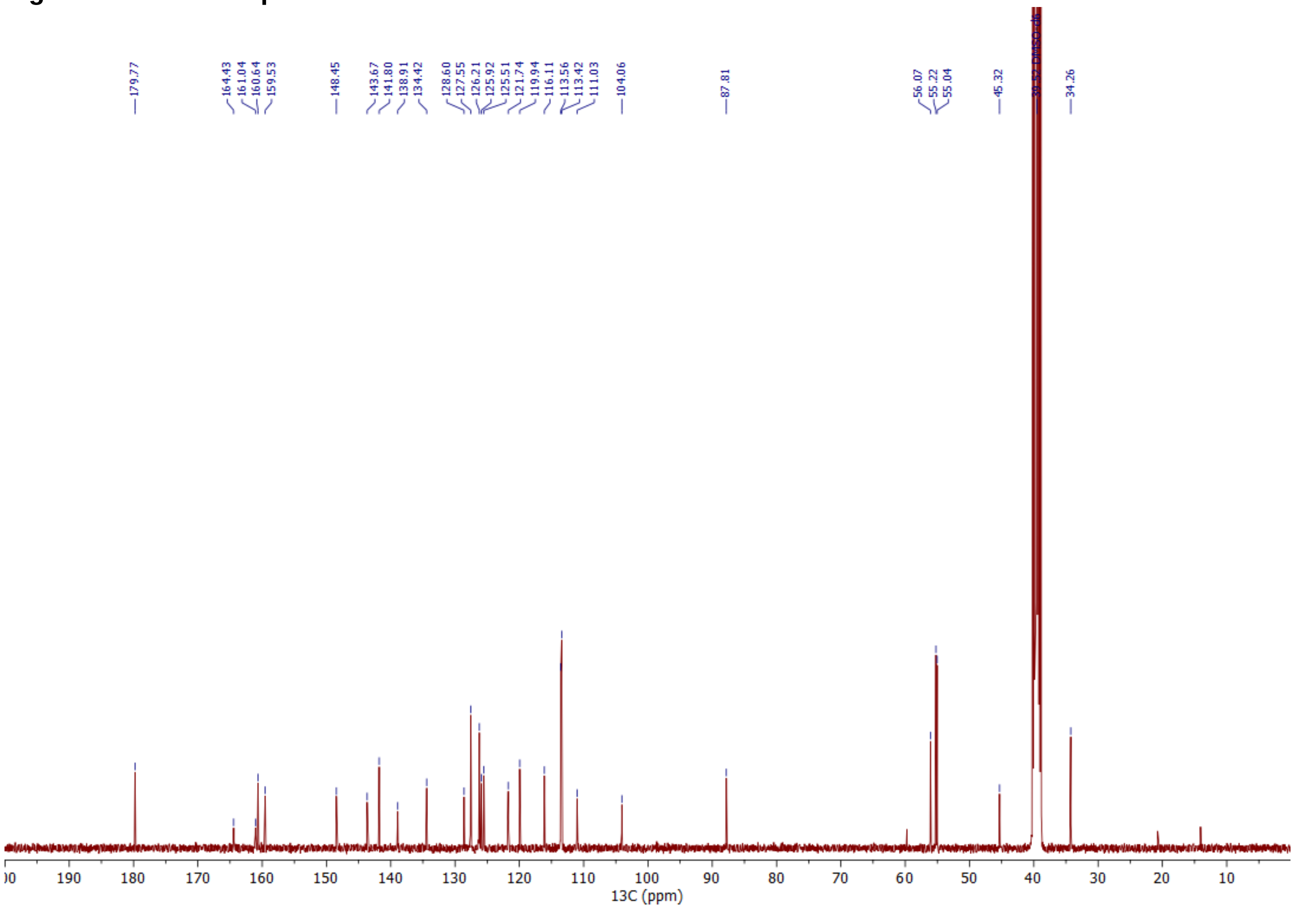

Figure S10. ^{13}C NMR spectrum of 7e

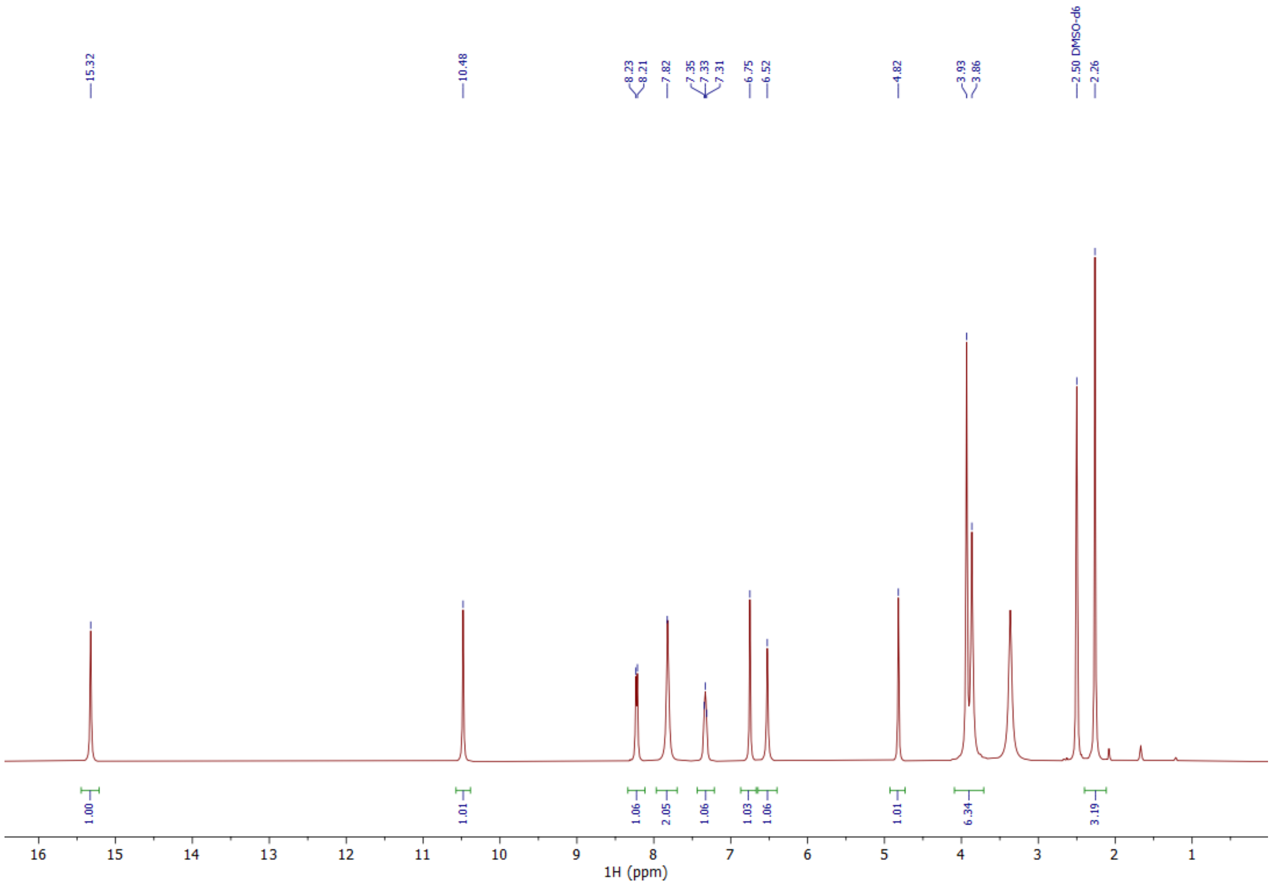

Figure S11. ^{1}H NMR spectrum of 7f

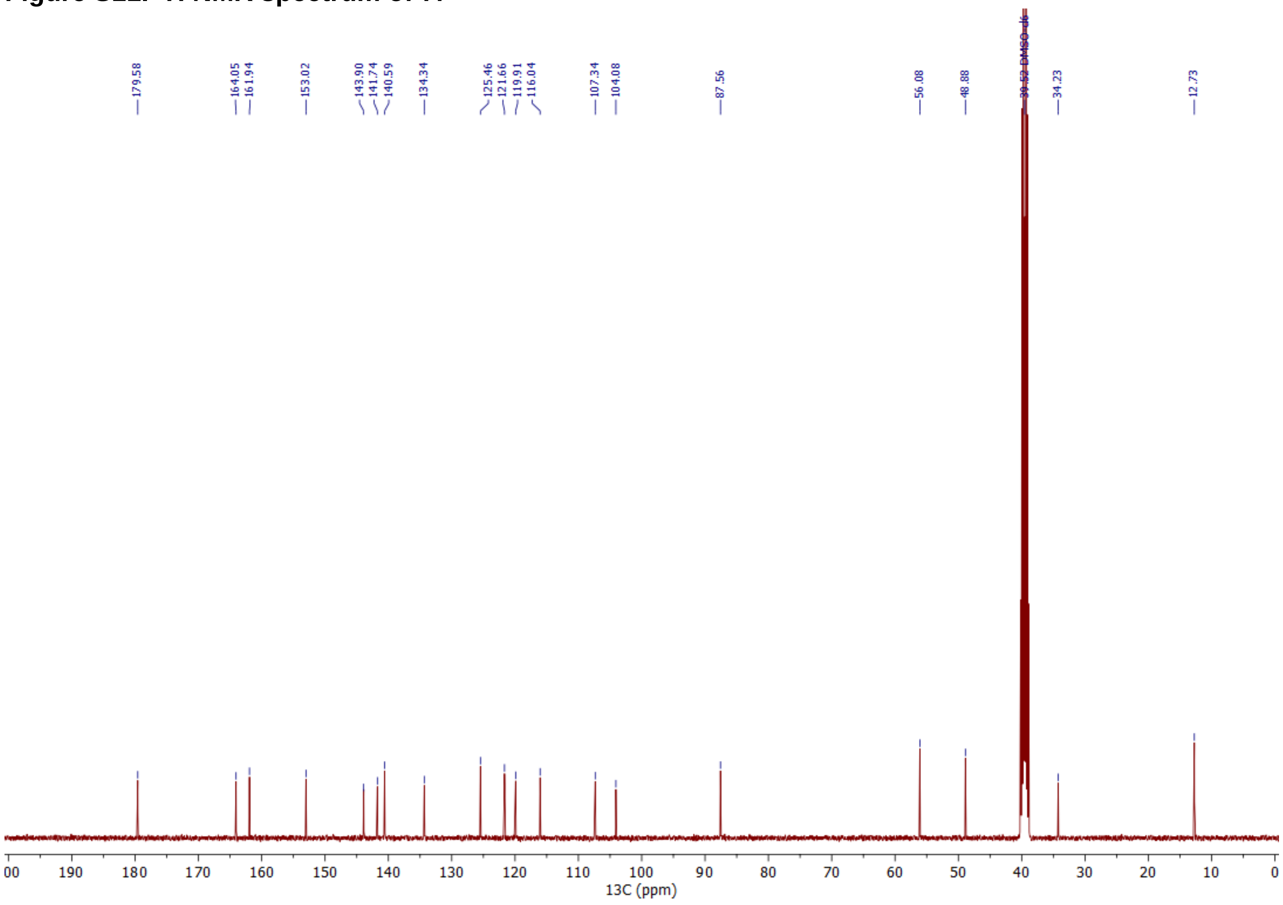

Figure S12. ^{13}C NMR spectrum of 7f

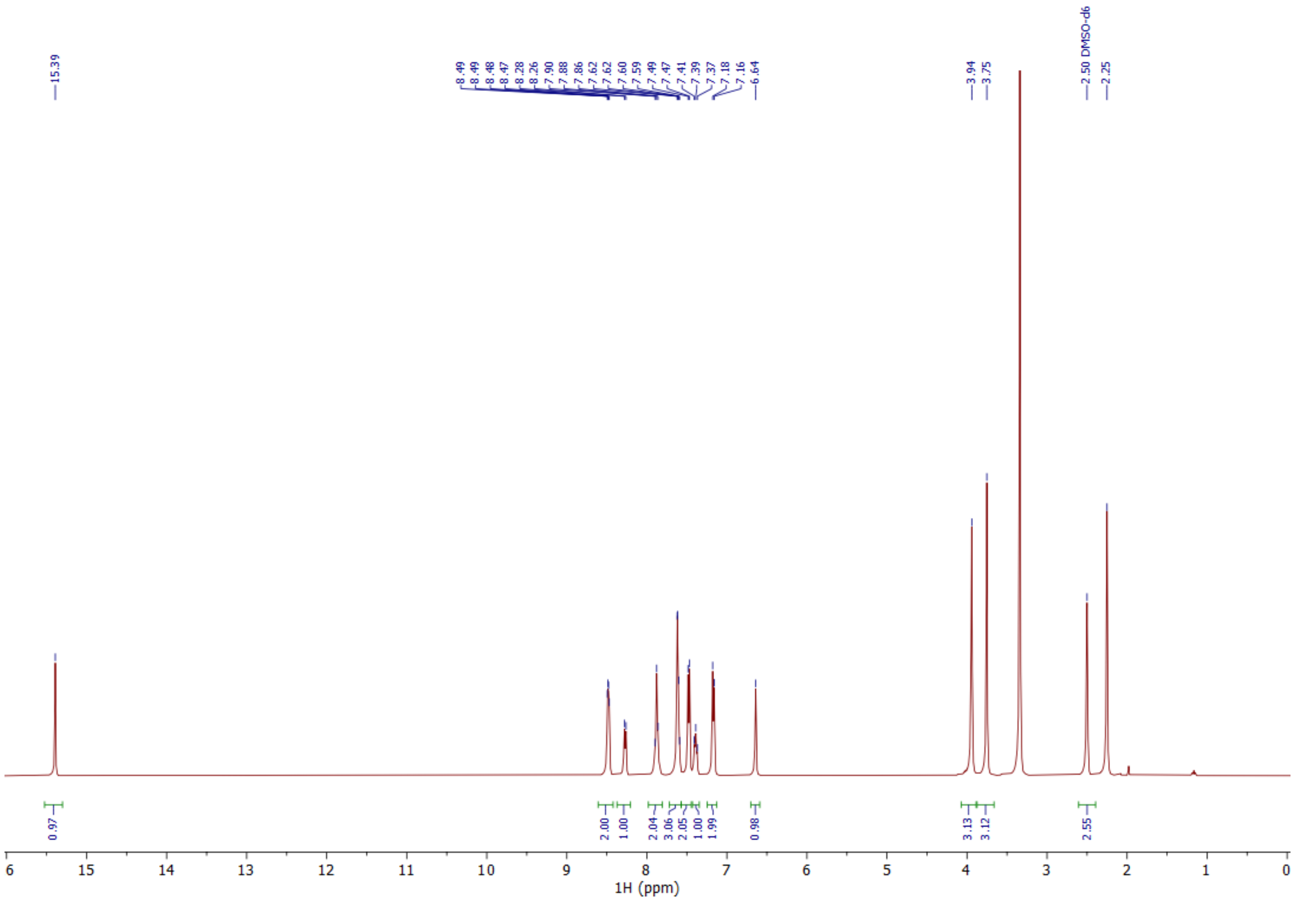

Figure S13. ^{1}**H NMR spectrum of 9b**

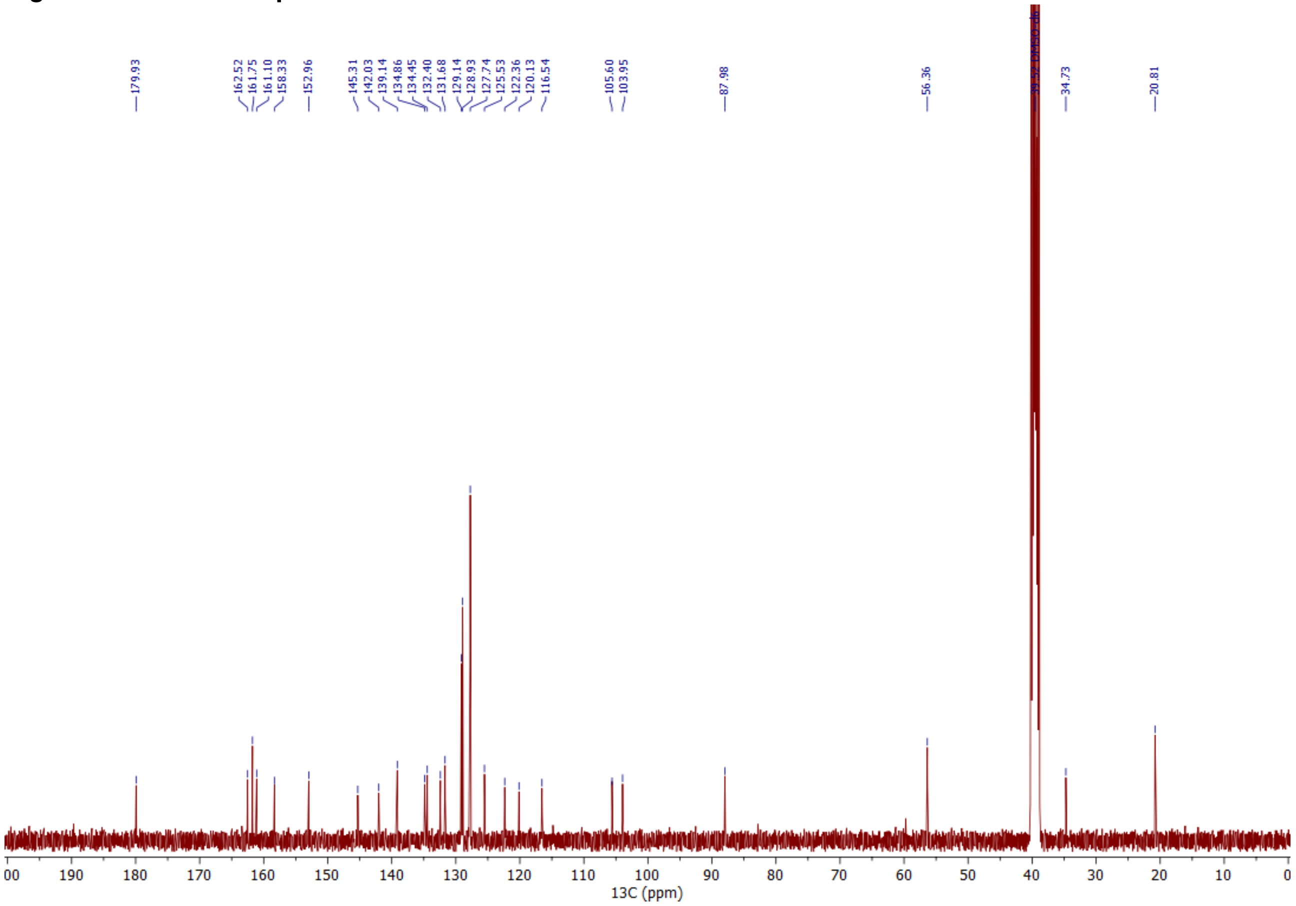

Figure S14. ^{13}**C NMR spectrum of 9b**

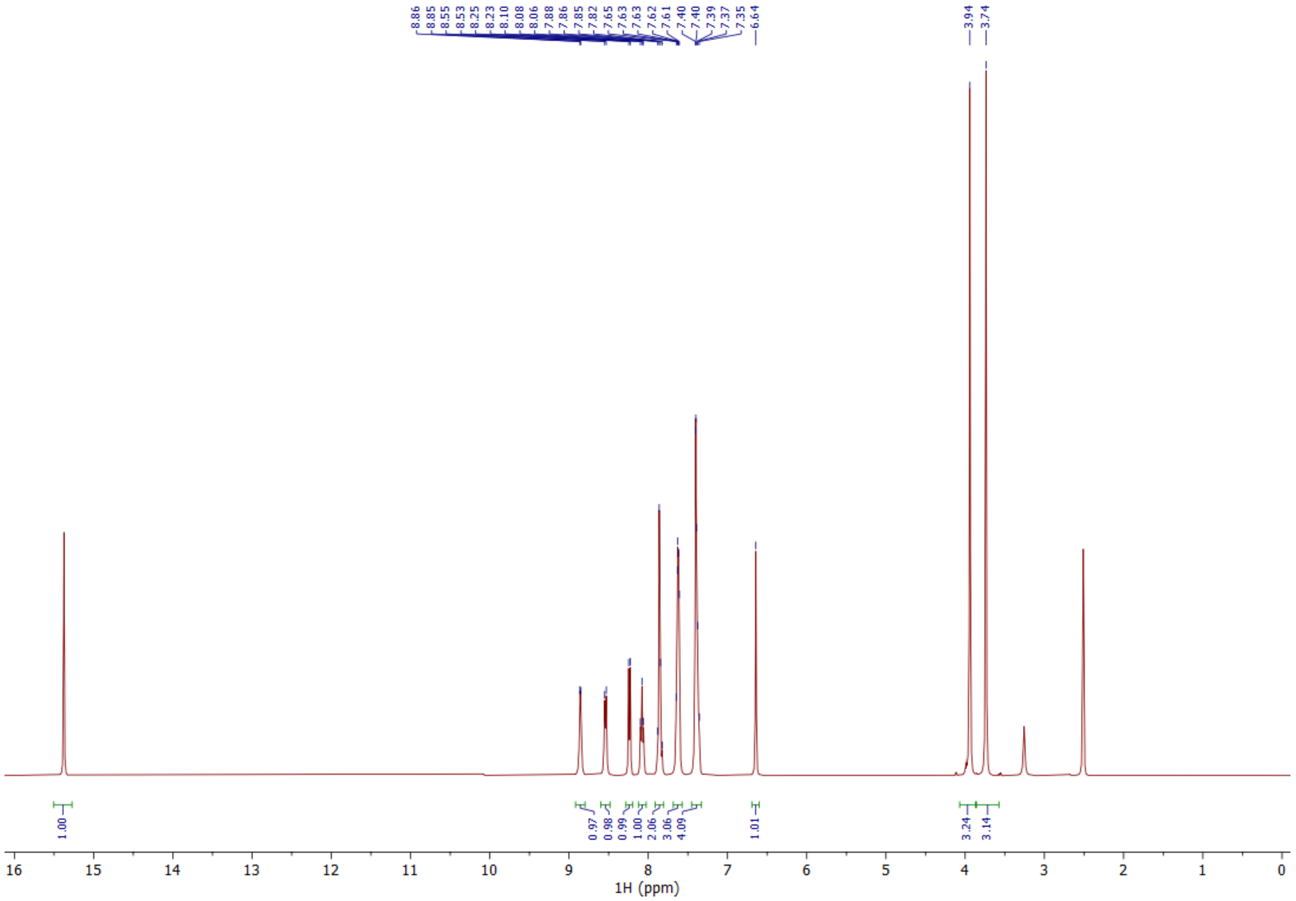

Figure S15. ^{1}**H NMR spectrum of 9c**

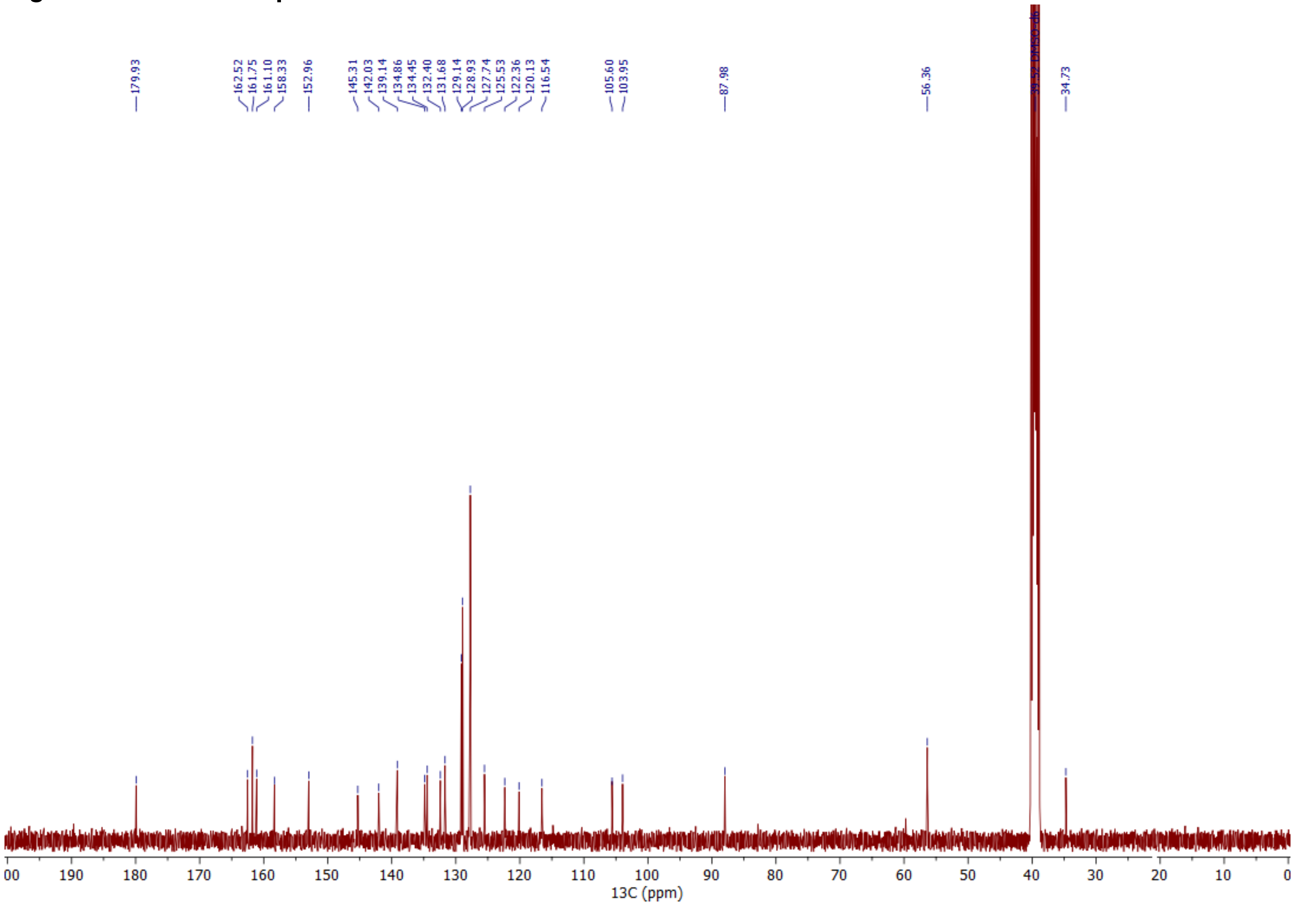

Figure S16. ^{13}**C NMR spectrum of 9c**

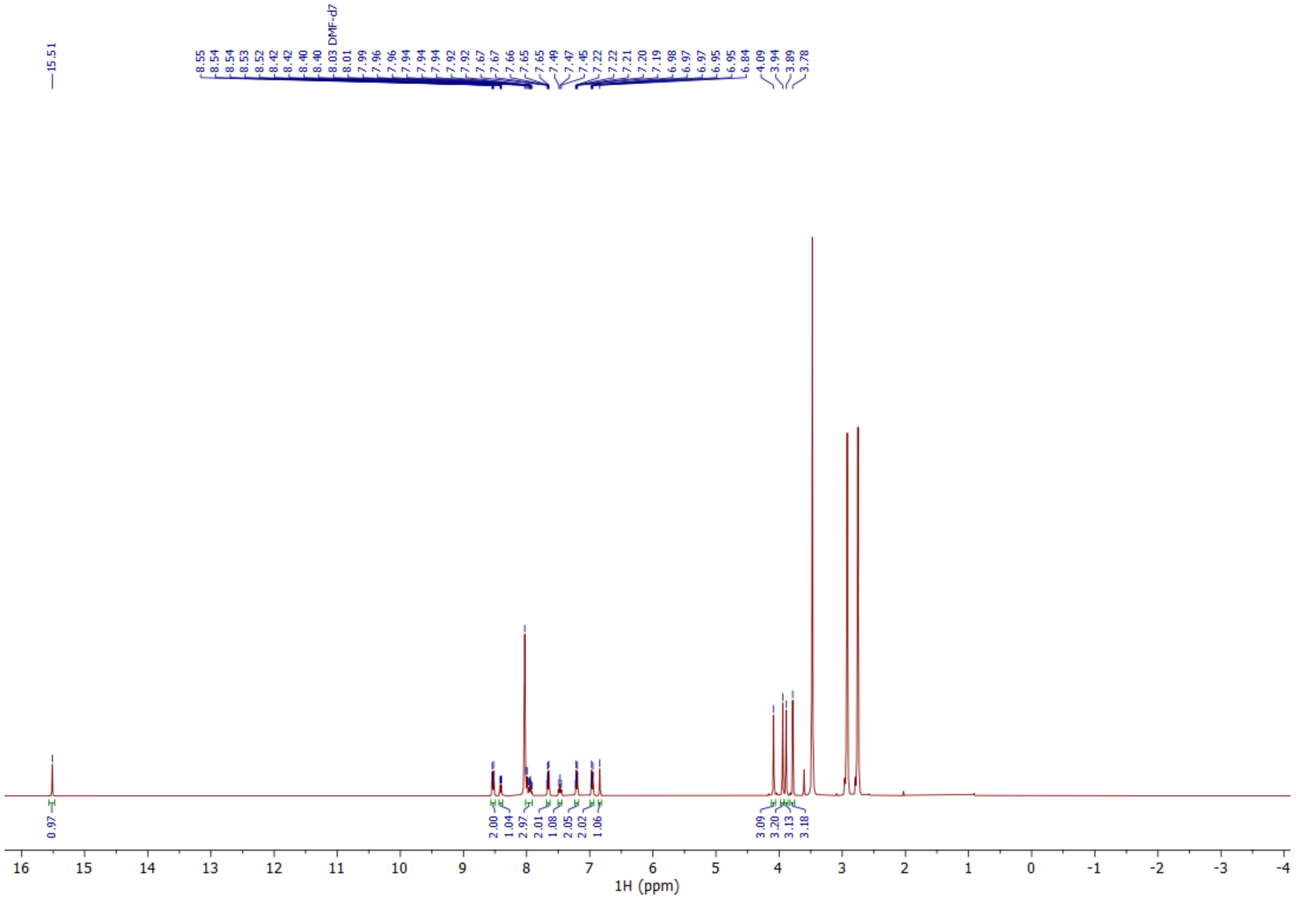

Figure S17. ^{1}H NMR spectrum of 9e

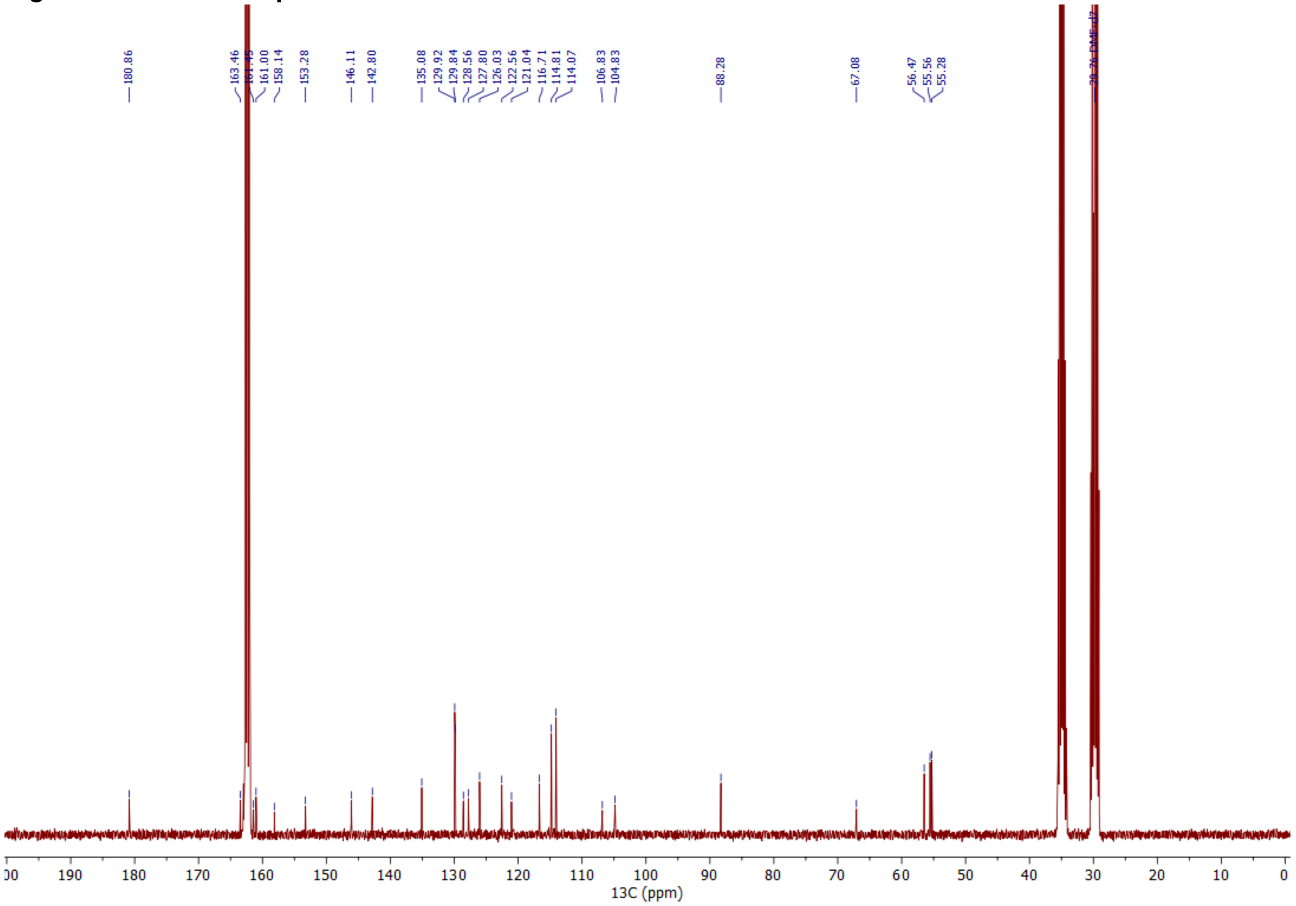

Figure S18. ^{13}C NMR spectrum of 9e

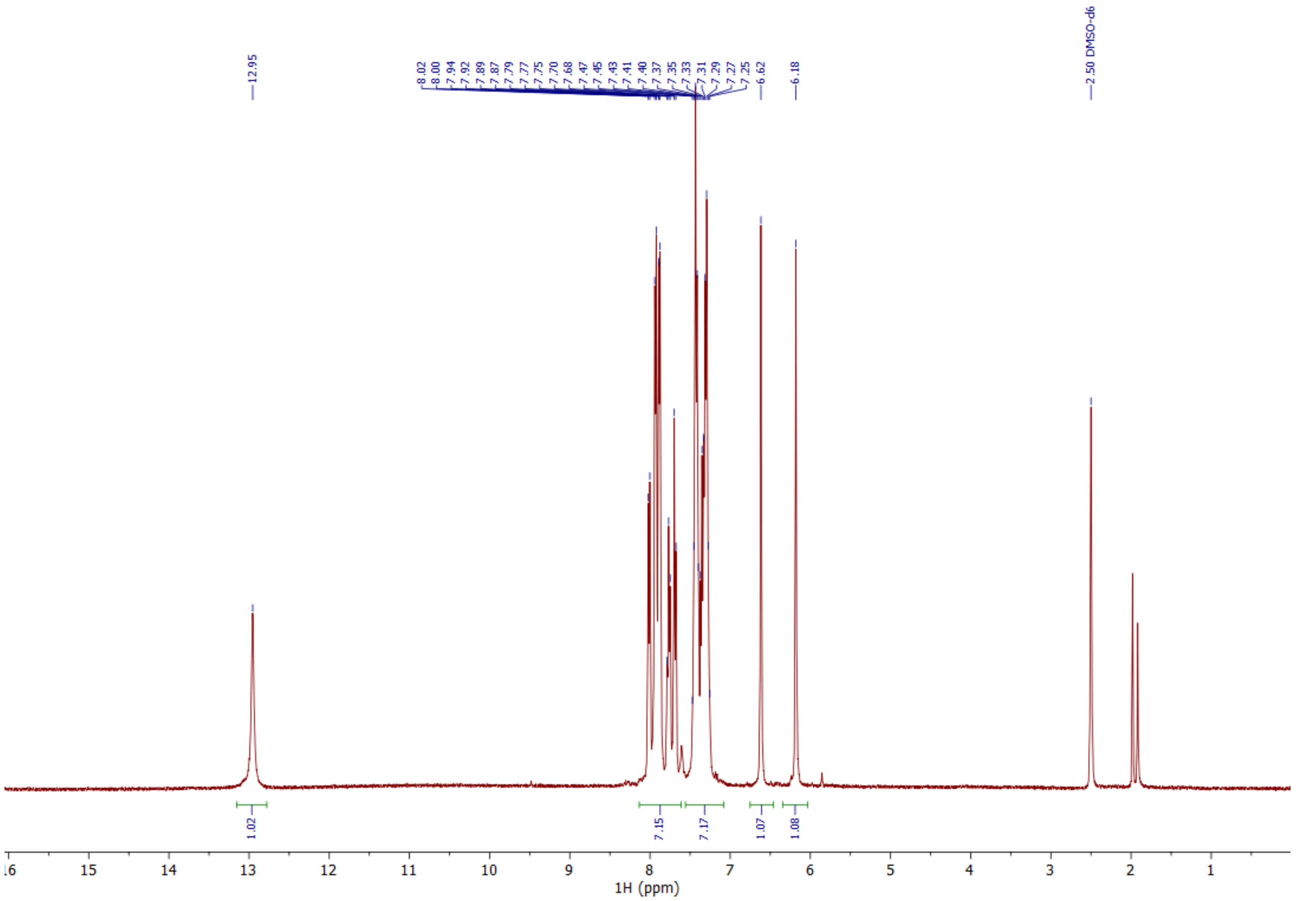

Figure S19. **^{1}H NMR spectrum of 12a**

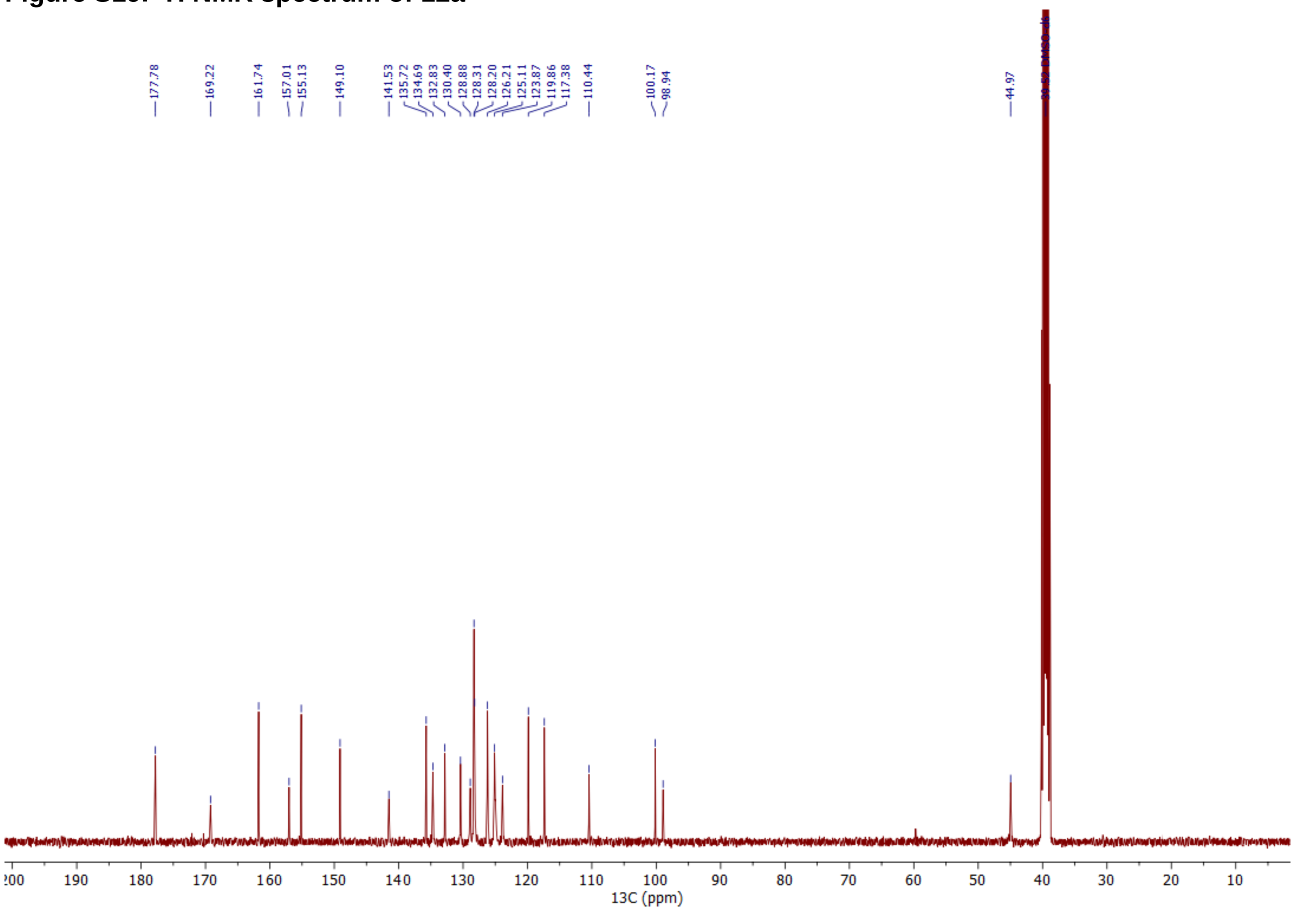

Figure S20. **^{13}C NMR spectrum of 12a**

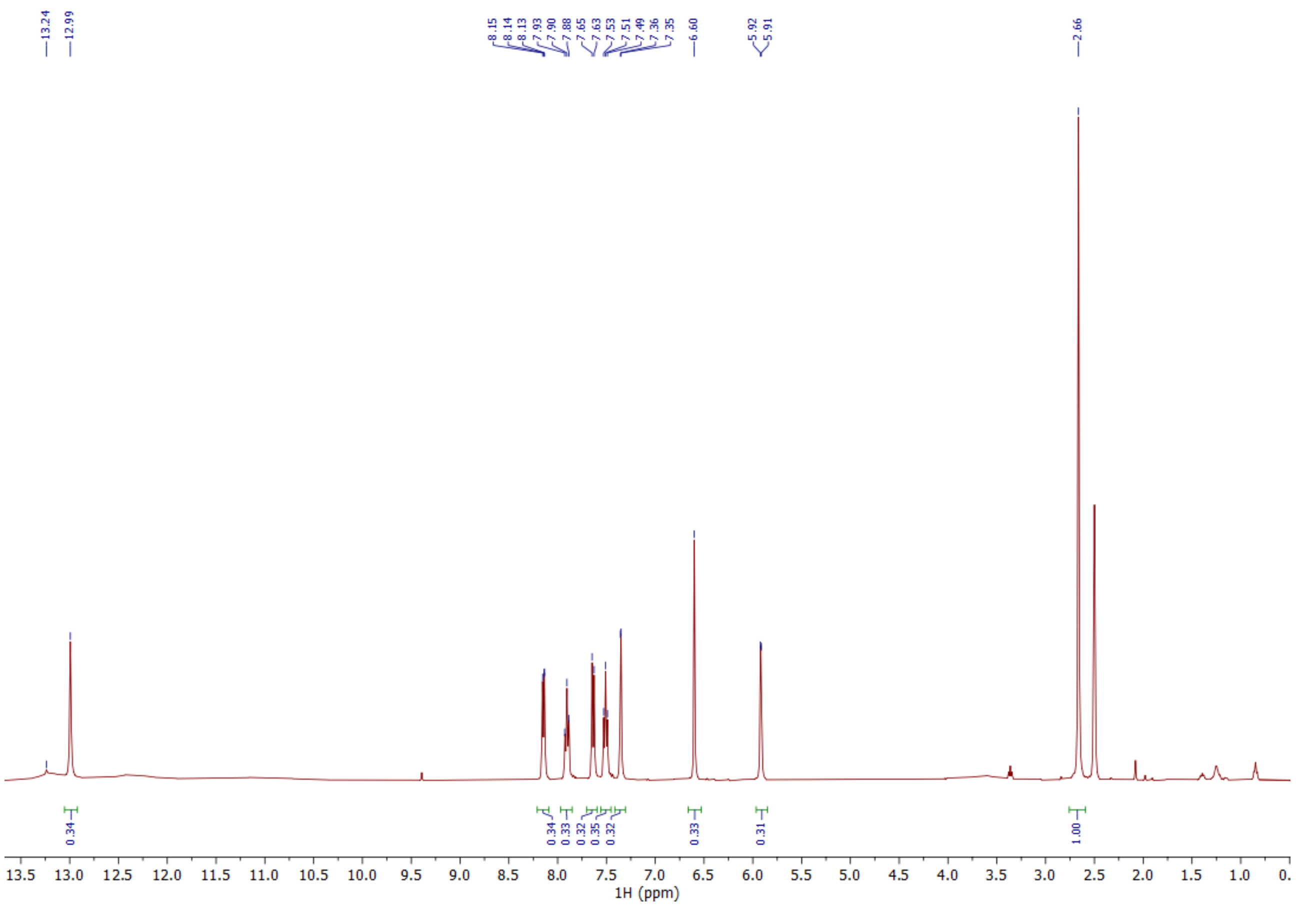

Figure S21. ^{1}H NMR spectrum of 12b

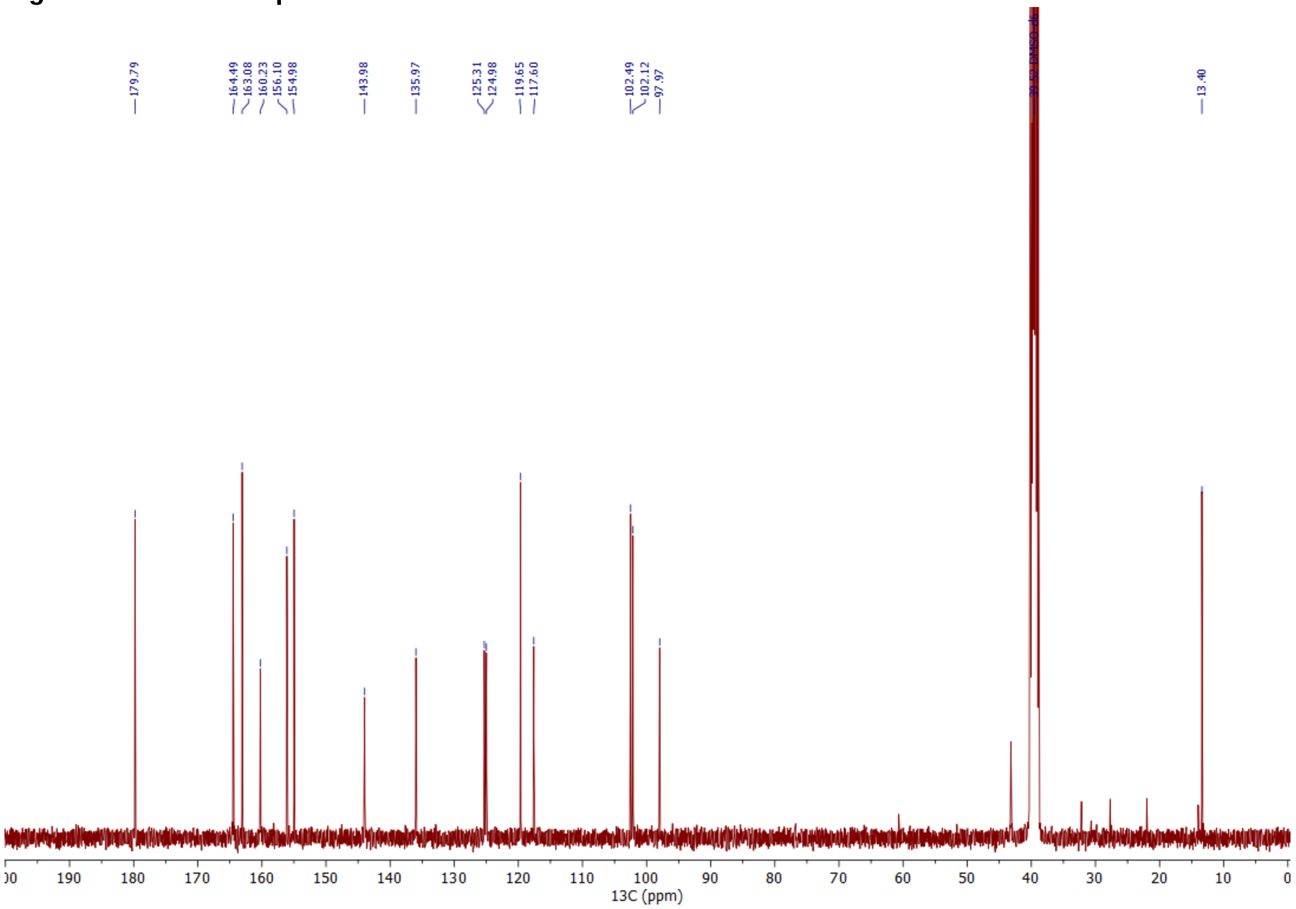

Figure S22. ^{13}C NMR spectrum of 12b

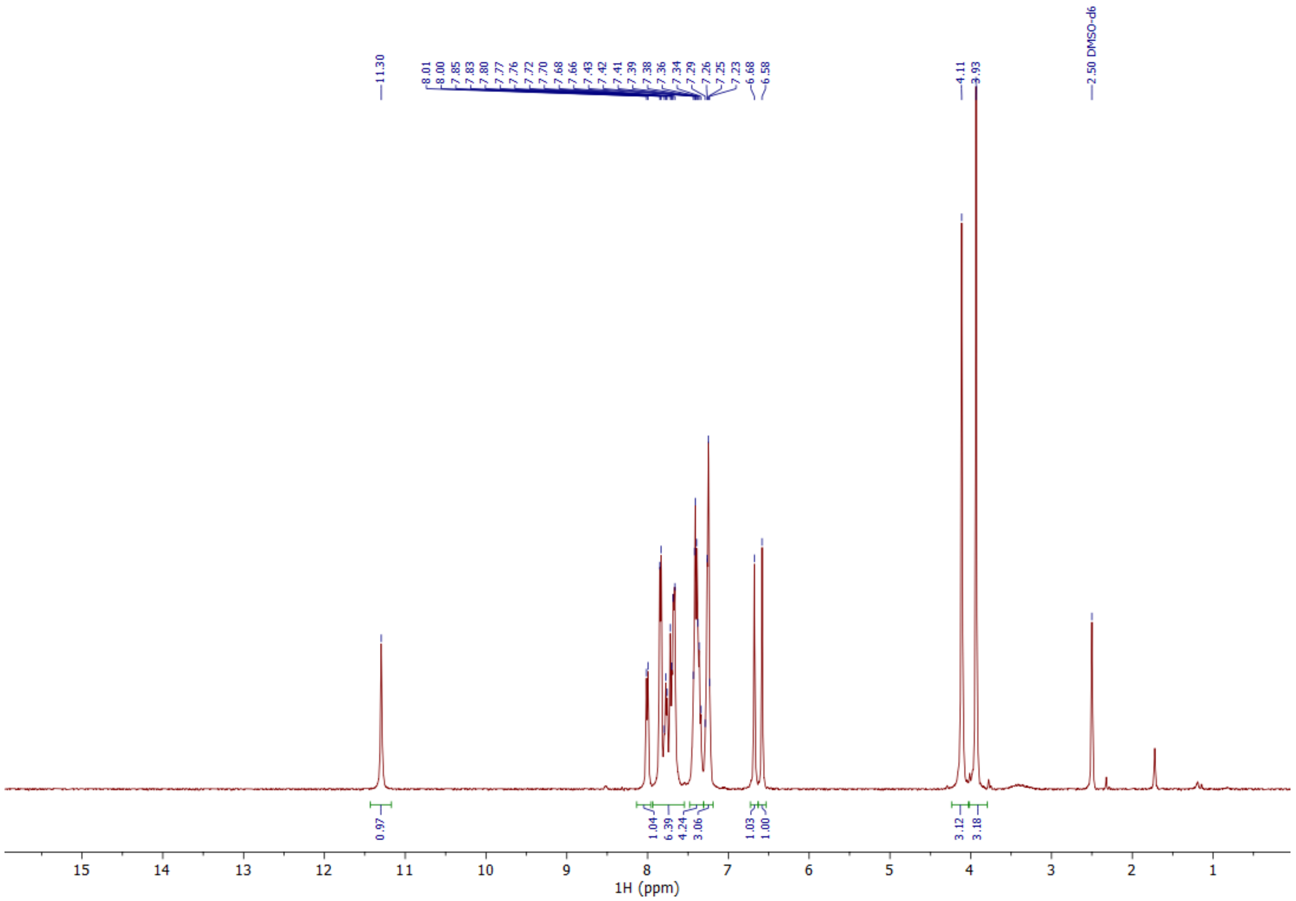

Figure S23. ^{1}H NMR spectrum of 14a

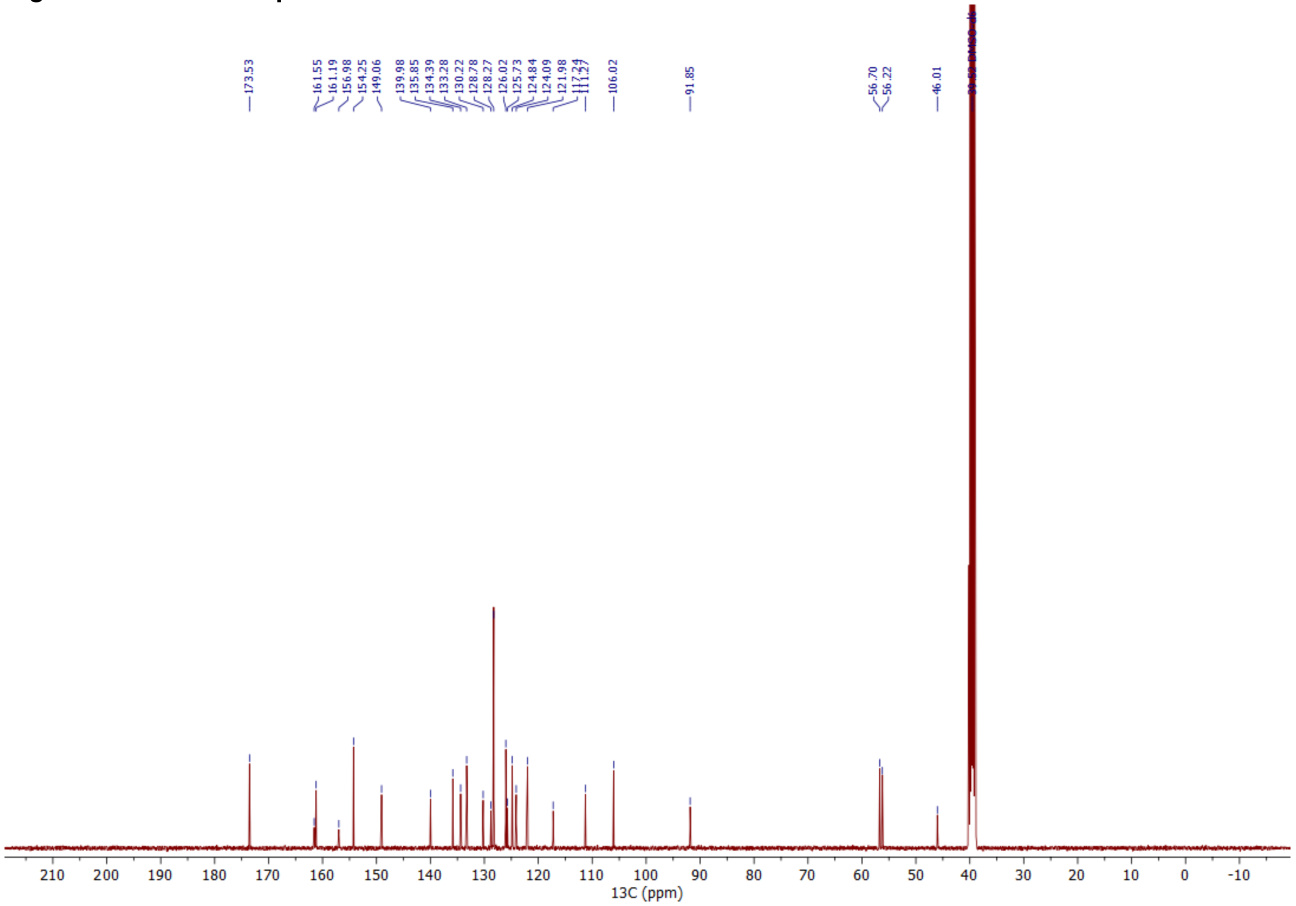

Figure S24. ^{13}C NMR spectrum of 14a

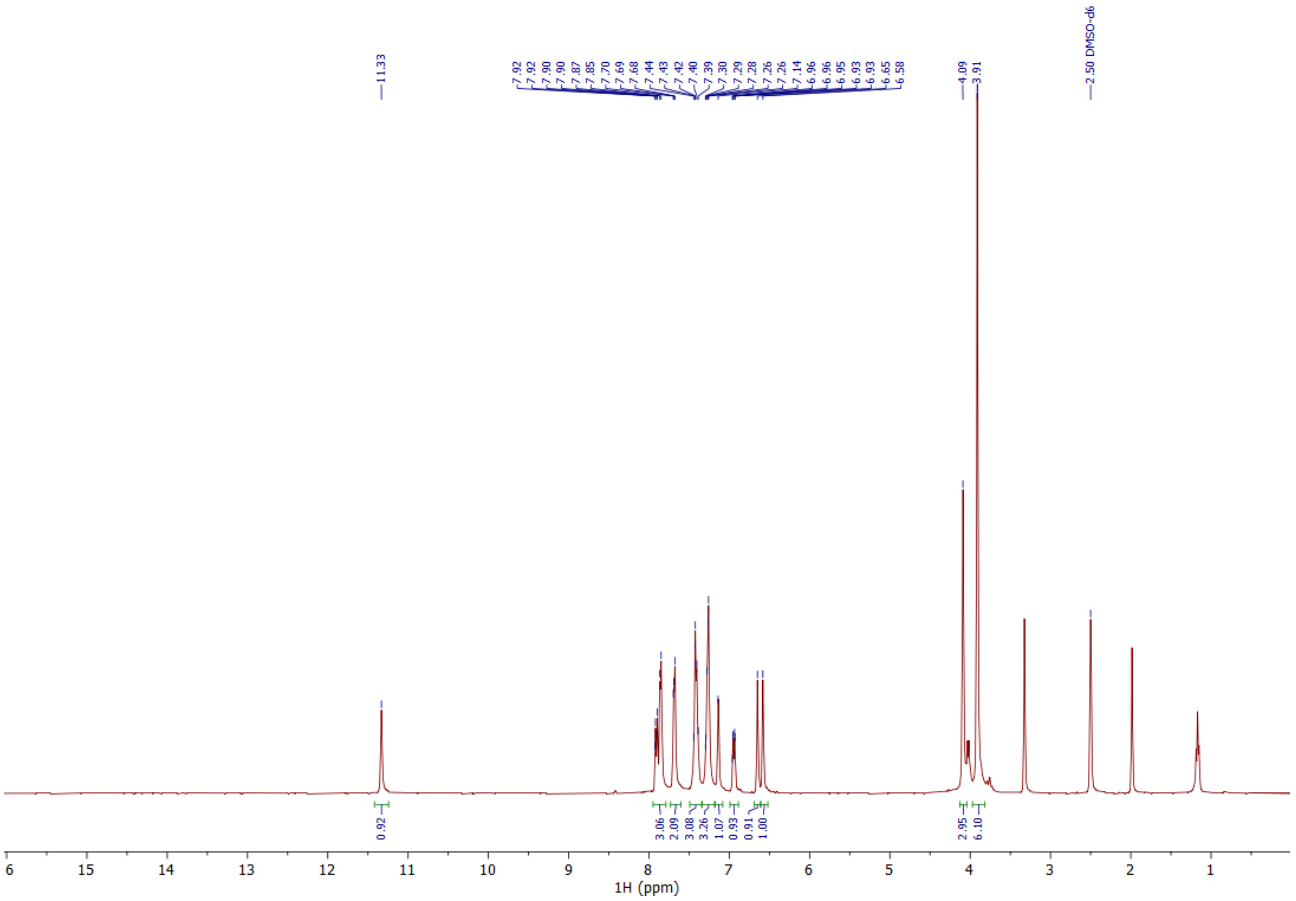

Figure S25. ¹H NMR spectrum of 14b

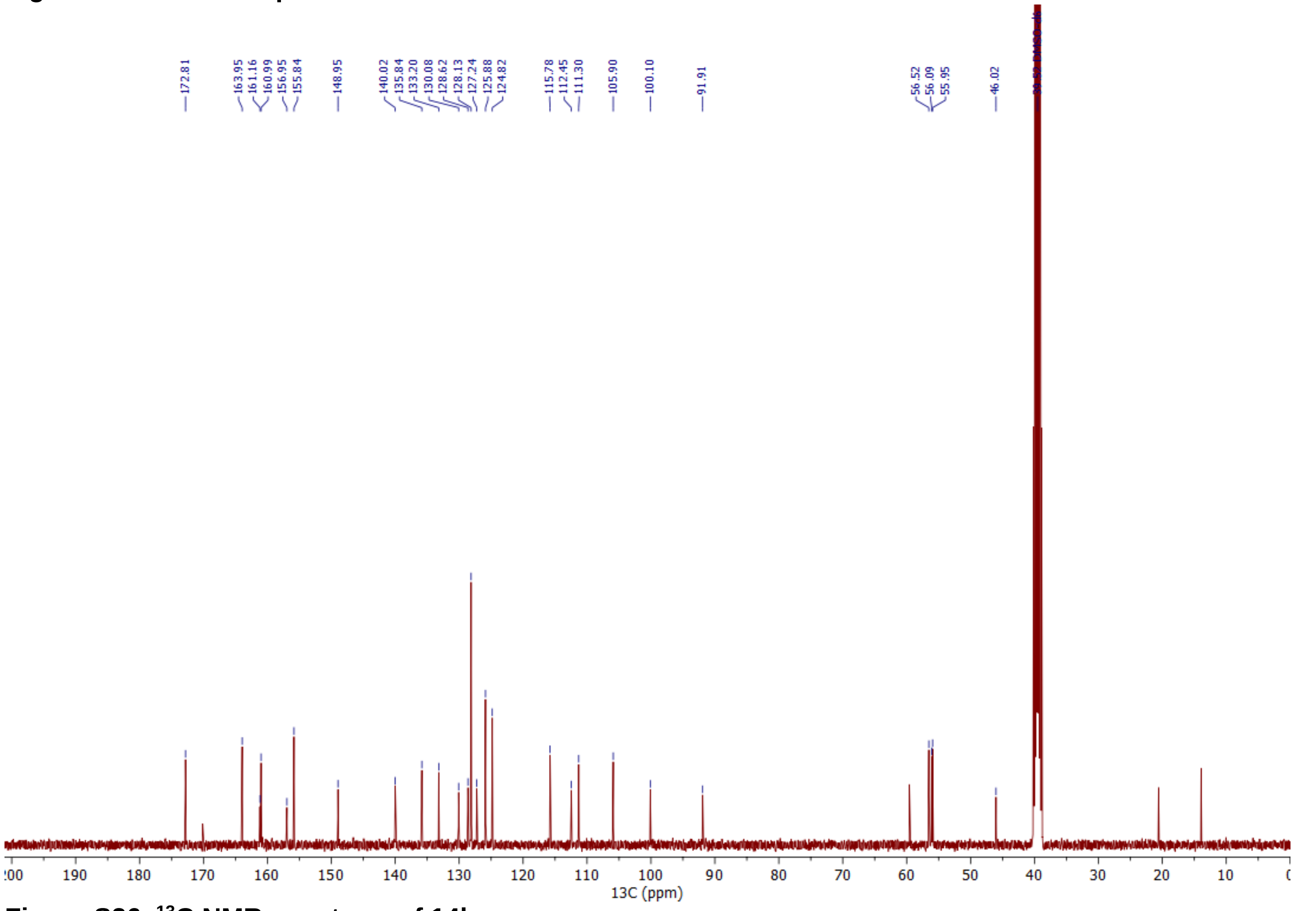

Figure S26. ¹³C NMR spectrum of 14b

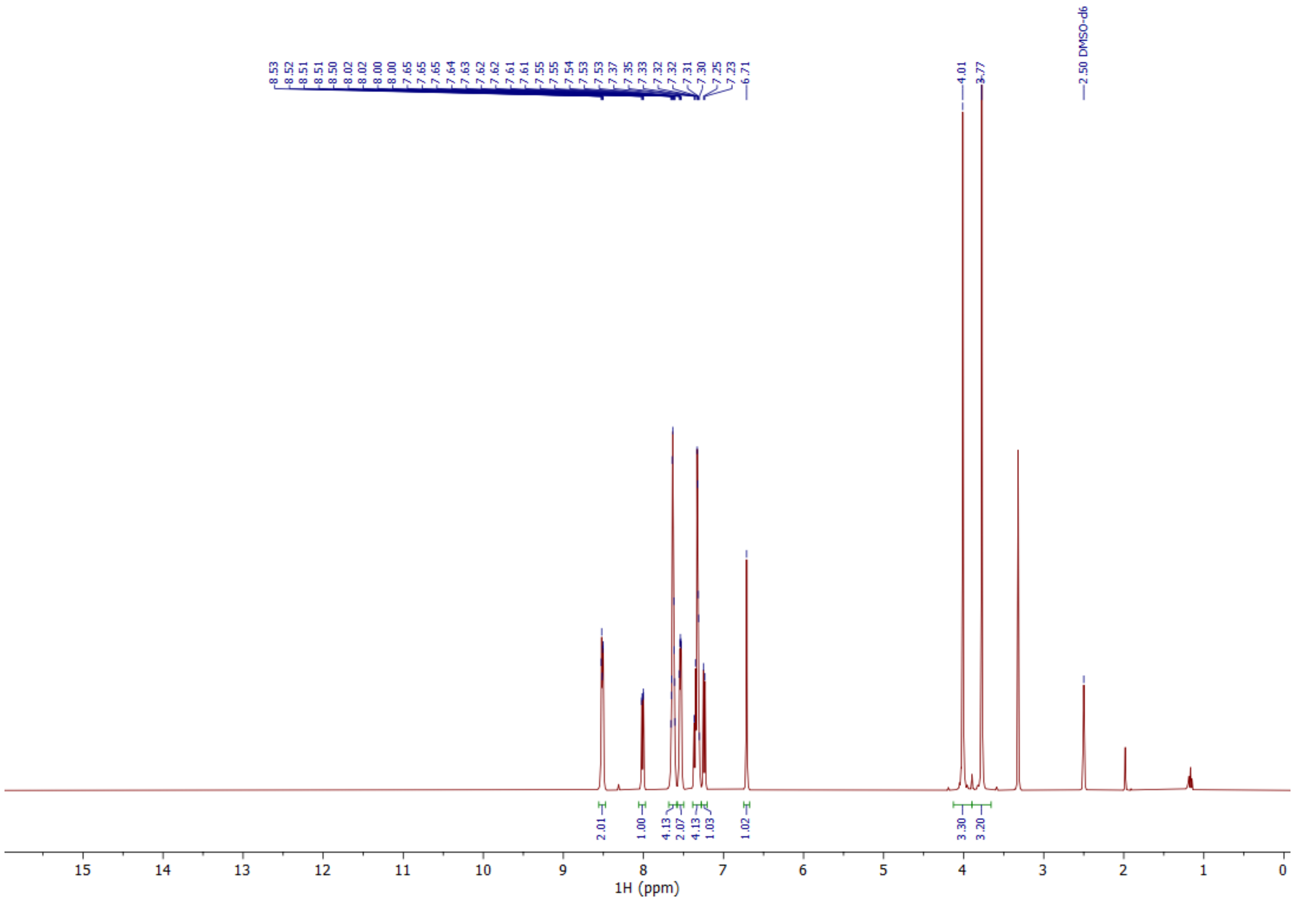

Figure S27. [1]**H NMR spectrum of 15a**

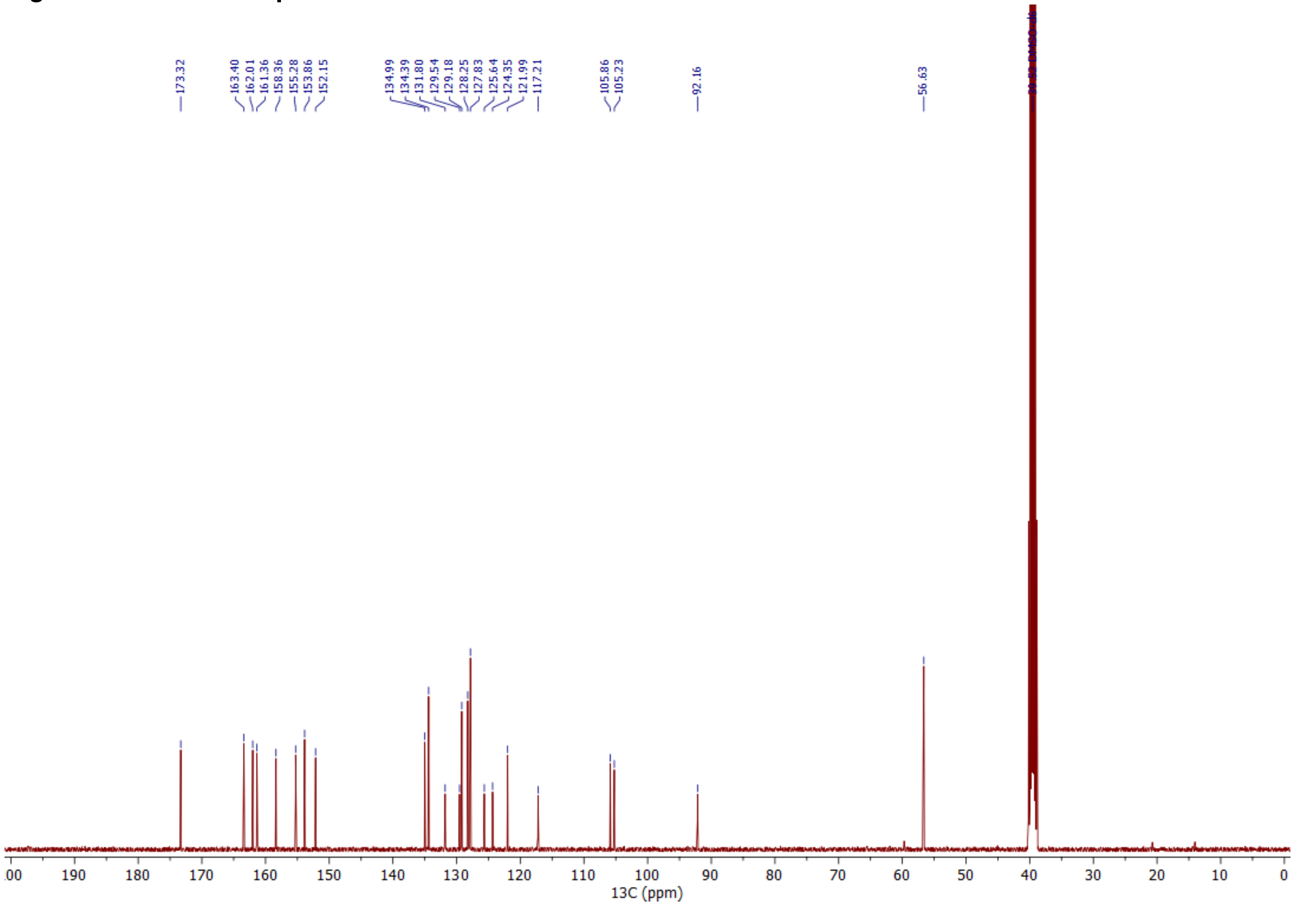

Figure S28. [13]**C NMR spectrum of 15a**

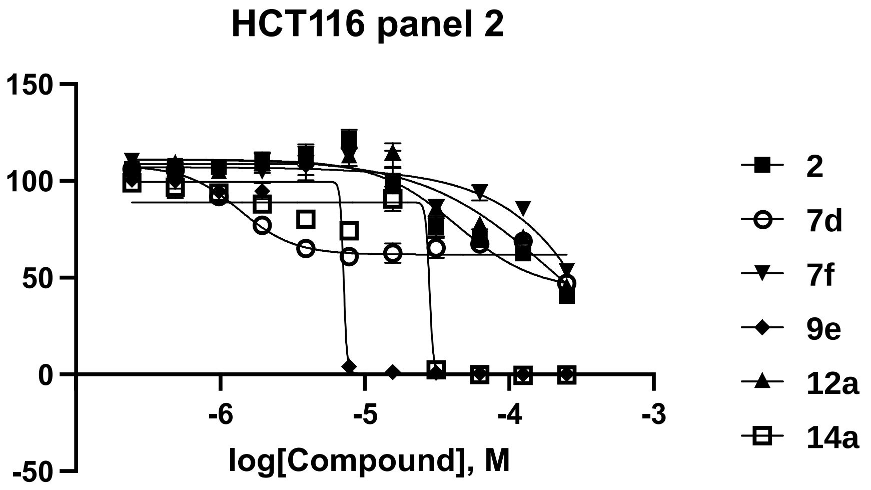

Figure S29. HCT116 cell survival curve for compounds 2, 7d, 7f, 9e, 12a and 14a

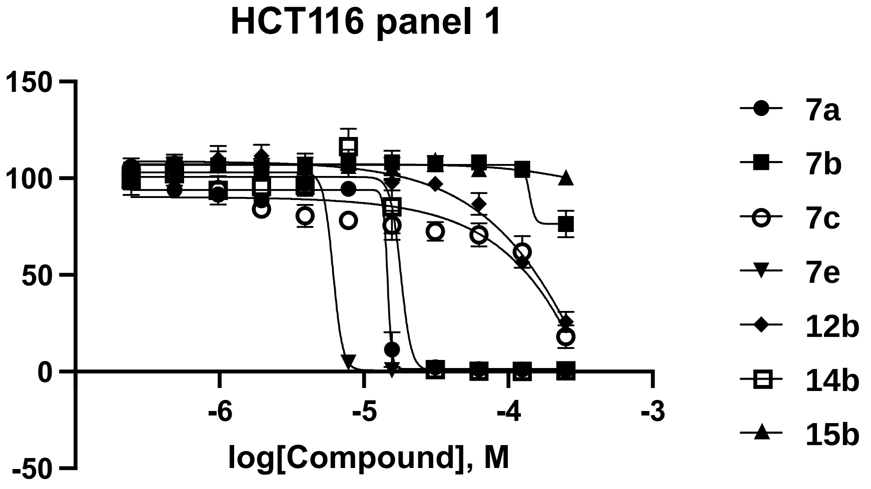

Figure S30. HCT116 cell survival curve for compounds 7a-c,e, 12b, 14b, 15b
